# The c-Abl-RACK1-FAK signaling axis promotes renal fibrosis in mice through regulating fibroblast-myofibroblast transition

**DOI:** 10.1186/s12964-024-01603-z

**Published:** 2024-04-30

**Authors:** Qianyi Bao, Anyu Wang, Wenxuan Hong, Yushu Wang, Baojie Li, Lin He, Xiaodong Yuan, Gang Ma

**Affiliations:** 1 School of Medicine, Southeast University, 87 Ding Jiaqiao Rd, Nanjing, 210009 P.R. China; 2 Bio-X Institutes, Key Laboratory for the Genetics of Developmental and Neuropsychiatric Disorders (Ministry of Education), Shanghai Jiao Tong University, Shanghai, P.R. China; 3 Department of Cardiology, Shanghai Institute of Cardiovascular Diseases, Zhongshan Hospital, Fudan University, Shanghai, P.R. China; 4 Department of Urology, Renji Hospital, Shanghai Jiao Tong University, School of Medicine, Shanghai, P.R. China

**Keywords:** c-Abl, Renal fibrosis, RACK1, Fibroblast-myofibroblast transition, Focal adhesion

## Abstract

**Background:**

Renal fibrosis is a prevalent manifestation of chronic kidney disease (CKD), and effective treatments for this disease are currently lacking. Myofibroblasts, which originate from interstitial fibroblasts, aggregate in the renal interstitium, leading to significant accumulation of extracellular matrix and impairment of renal function. The nonreceptor tyrosine kinase c-Abl (encoded by the Abl1 gene) has been implicated in the development of renal fibrosis. However, the precise role of c-Abl in this process and its involvement in fibroblast-myofibroblast transition (FMT) remain poorly understood.

**Methods:**

To investigate the effect of c-Abl in FMT during renal fibrosis, we investigated the expression of c-Abl in fibrotic renal tissues of patients with CKD and mouse models. We studied the phenotypic changes in fibroblast or myofibroblast-specific c-Abl conditional knockout mice. We explored the potential targets of c-Abl in NRK-49F fibroblasts.

**Results:**

In this study, fibrotic mouse and cell models demonstrated that c-Abl deficiency in fibroblasts mitigated fibrosis by suppressing fibroblast activation, fibroblast-myofibroblast transition, and extracellular matrix deposition. Mechanistically, c-Abl maintains the stability of the RACK1 protein, which serves as a scaffold for proteins such as c-Abl and focal adhesion kinase at focal adhesions, driving fibroblast activation and differentiation during renal fibrosis. Moreover, specifically targeting c-Abl deletion in renal myofibroblasts could prove beneficial in established kidney fibrosis by reducing RACK1 expression and diminishing the extent of fibrosis.

**Conclusions:**

Our findings suggest that c-Abl plays a pathogenic role in interstitial fibrosis through the regulation of RACK1 protein stabilization and myofibroblast differentiation, suggesting a promising strategy for the treatment of CKD.

**Supplementary Information:**

The online version contains supplementary material available at 10.1186/s12964-024-01603-z.

## Background

Renal fibrosis is characterized by the excessive accumulation of pathological extracellular matrix (ECM) in the interstitial space between tubules and peritubular capillaries, disrupting their normal ability to eliminate toxins and provide nutrients [1]. Chronic kidney disease (CKD), the leading cause of renal fibrosis, has a reported prevalence rate of approximately 8.6% for males and 9.6% for females older than the age of 20 years in high-income countries [2]. Unfortunately, currently available medications only offer limited disease progression-slowing benefits, underscoring the urgent need to improve the understanding of the mechanisms underlying CKD and renal fibrosis [3].

Fibroblast-myofibroblast transition (FMT) is a crucial stage in the development of fibrosis due to the pivotal role of myofibroblasts in the synthesis of α-SMA and ECM. α-SMA serves as the most reliable marker for identifying myofibroblasts, as it constitutes the primary component of myofilament bundles known as stress fibers. These stress fibers are anchored to focal adhesions, which connect the ECM to the actin cytoskeleton [4]. By means of focal adhesions, stress fibers respond to mechanical tension within the ECM and transmit contractile forces. The tension or contractility of stress fibers enables the conversion of mechanical signals into biochemical cues, thereby playing a crucial role in the maturation and dynamics of focal adhesions [5]. Excessive maturation of focal adhesions, a hallmark feature of lesional fibroblasts, can facilitate the formation of stress fibers and enhance fibroblast adhesion to the matrix. This process relies on integrin binding and the activation of focal adhesion kinase (FAK) [6]. Ultimately, in conjunction with certain cytokines, such as transforming growth factor-beta 1 (TGF-β1), cytoskeletal reorganization and myofibroblast differentiation are triggered [1]. Nonetheless, the specific molecular mechanisms governing FMT have largely not been identified.

TGF-β1 is a key regulator that drives myofibroblast differentiation during fibrosis [7]. Targeting the TGF-β-regulated intracellular pathways is a major focus of emerging antifibrotic therapies [8]. Considering the essential physiological roles of canonical TGF-β1 signaling in cell proliferation, differentiation, tissue homeostasis, and the immune response [9], direct targeting of TGF-β1 could have potential adverse effects, including liver [10] and cardiac toxicity [11, 12]. Noncanonical TGF-β signaling pathways are being explored as therapeutic targets in organ fibrosis. c-Abl, encoded by ABL proto-oncogene 1, is a member of the Abelson family of nonreceptor tyrosine kinases involved in noncanonical TGF-β signaling. Recent studies have shown that silencing c-Abl may facilitate the elimination of myofibroblasts from wound lesions [13]. A c-Abl-MRTF-A positive feedback loop contributes to hepatic stellate cell activation and liver fibrosis [14]. Imatinib mesylate, a small molecule inhibitor of c-Abl, has been found to have an effect on systemic sclerosis fibroblasts in vitro [15]. However, whether c-Abl is involved in FMT occurrence during renal fibrosis and whether it regulates focal adhesion maturation and microfilament formation are unclear.

The present study demonstrated significant upregulation of c-Abl in fibrotic kidneys in both human and mouse samples, specifically in the ECM-expressing PDGFRα^+^ fibroblasts. Notably, a fibroblast-specific deletion of c-Abl resulted in a marked reduction in renal fibrosis in mouse models of unilateral ureteral obstruction (UUO). Our findings indicate that excessive c-Abl exacerbates fibrogenesis by promoting fibroblast activation and ECM production. Mechanistically, we discovered that c-Abl promotes the maturation of focal adhesions and the assembly of stress fibers by stabilizing the expression of the receptor for activated C-kinase 1 (RACK1) protein, a member of the tryptophan-aspartate repeat (WD repeat) family and a classic scaffold protein [16]. Finally, our results demonstrated that myofibroblast-specific deletion of c-Abl effectively mitigates the development and progression of kidney fibrosis in mice, suggesting that targeting c-Abl could serve as a novel and potent approach for the treatment of renal fibrosis and preservation of kidney function.

## Methods

### Human renal biopsy samples

Human renal samples were obtained from clinical kidney biopsies performed on patients with uremia and healthy kidney donors. For CKD group, we analyzed the nontumor sites of the kidneys from 9 patients who underwent nephrectomy (total nephrectomy). Healthy kidneys from 9 cases without CKD were analyzed as the control group. The human study was approved by the Institutional Review Board of the Ethics Committee of Renji Hospital, Shanghai Jiao Tong University The ethics approval number is No. LY2023-032-B.

### Mouse models and generation of animals with renal fibrosis

All animal experiments in this study were conducted strictly in accordance with the National Research Council Guide for the Care and Use of Laboratory Animals with protocols approved by the Institutional Animal Care and Use Committee of our school (A2023086) and ARRIVE guidelines. Strains used in this study include *PDGFRα-CreER* mice (Jackson lab., stock No. 018280), *α-SMA-CreER* mice [17], and *c-Abl*^*loxP/loxP*^ mice [18]. Breeding details and strain genetics are outlined in the Supplementary Methods.

### Cell-based assays

**Rat renal fibroblast line NRK-49F** NRK-49F cells were purchased from the American Type Culture Collection and cultured in DMEM containing 10% FBS and 1% penicillin‒streptomycin at 37 °C under 5% CO2.

**TGF-β1 stimulation** NRK-49F cells were incubated with 10 ng/ml TGF-β1 (PeproTech, 100–21) for 24 h, after which the following experiments were performed.

**Inhibitor experiments** For the analysis of microtubules, the microtubules were stabilized by the addition of 0.5 μmol/L paclitaxel (TargetMol Chemicals, T0968) and depolymerized by the addition of 0.1 μmol/L vinblastine (TargetMol Chemicals, T6721). For the analysis of protein stability, the protein synthesis inhibitor cycloheximide at 20 μg/mL (MedChemExpress, HY-12320), the lysosome inhibitor chloroquine at 15 μmol/L (TargetMol Chemicals, T8689), bafilomycin A1 at 5 nmol/L (TargetMol Chemicals, T6740), or the proteasome inhibitor MG132 at 5 μmol/L (TargetMol Chemicals, T2154) were added to the cell culture medium.

**Lentivirus infection** To construct stable NRK-49F cell lines for the overexpression or knockdown of c-Abl and overexpression of RACK1, a lentivirus carrying the corresponding genes was applied to the cells combined with polybrene (8 µg/ml) (Yeasen Biotechnology, 40804ES76) for 12 h. Then, the medium was replaced with fresh medium, and stable cell clones were screened using blasticidin S at 20 µg/ml (Beyotime Biotechnology, ST018) for 3 generations to ensure overexpression or knockdown efficiency.

**RACK1 knockdown** The siRNAs for rat RACK1 were designed and synthesized by D-Nano Therapeutics Company (Beijing, China). CALNPTM siRNA Transfection Reagent (D-Nano Therapeutics) was used to transfect siRNA or negative control cells at 10 nmol/L according to the manufacturer's instructions. The sequences of the siRNAs for RACK1 were as follows: GCAAGCACCUUUACACAUUTT, Antisense: AAUGUGUAAAGGUGCUUGCTT. The sequences of the siRNAs for the control were as follows: UUCUCCGAACGUGUCACGUTT, Antisense: ACGUGACACGUUCGGAGAATT.

### Histological analysis

Kidney tissues were fixed in 4% paraformaldehyde (PFA) overnight, embedded in paraffin, and subsequently cut into 4 μm sections. The sections were mounted onto glass slides, deparaffinized with xylene, dehydrated through a graded ethanol series, and stained with hematoxylin–eosin (Beyotime Biotechnology; C0105M), Sirius red (Solarbio Life Science, S8060) and Masson’s trichrome (Solarbio Life Science, G1340) to evaluate collagen deposition. The fibrotic area was quantified with ImageJ software.

### Immunohistochemical analysis

Immunohistochemical staining was performed with the peroxidase–antiperoxidase method. In brief, mouse kidney sections were retrieved by boiling them for 20 min in 10 mM citric acid solution (pH = 6). Endogenous peroxidase was blocked by incubation in 4% hydrogen peroxide. The sections were incubated at 4 °C overnight with the primary antibodies. On the next day, the tissue sections were washed and incubated with the corresponding secondary antibodies for 2 h at room temperature. The sections were then developed with DAB solution (ZSGB-BIO, ZLI-9019), counterstained with hematoxylin (Beyotime Biotechnolog, C0105S), and mounted with a neutral balsam medium (Solarbio LIFE SCIENCES, G8590). The staining area was measured by using ImageJ. The following antibodies were used: mouse anti-c-Abl (Sigma; A5844), rabbit anti-aSMA (Abcam; ab184705), rabbit anti-Col1a1 (Cell Signaling Technology; #72,026), rabbit anti-Vimentin (Abcam; ab92547), rabbit anti-F4/80 (Cell Signaling Technology; #70,076), mouse anti-RACK1 (BD Biosciences; 610,177). HRP-Goat Anti-Rabbit (H + L) (Proteintech, RGAR001), HRP-Goat Anti-mouse (H + L) (Proteintech, RGAM001).

### RNA extraction and qRT-PCR

Total RNA was extracted from cells and kidney tissue by using TRIzol (Vazyme; R401-01) in accordance with the manufacturer’s protocols. A HiScript III 1st Strand cDNA Synthesis Kit (+ gDNA wiper) (Vazyme; R312-01) was used for RNA reverse transcription. SYBR Green-based qRT-PCR (Vazyme; Q311-02) was performed as described in the protocol. The primer sequences used for qRT-PCR are listed in supplementary Table S2.

### Immunofluorescence analysis

**Tissue staining.** PFA-fixed kidneys were immersed in 30% sucrose overnight and embedded in OCT and were cut into 5 µm for frozen sections. The slides were washed with PBS and blocked in 10% FBS for 1 h, then incubated with the primary antibodies at 4 ℃ overnight and subsequently incubated with Alexa Fluor-conjugated secondary antibodies for 2 h at room temperature, then washed three times with PBS and finally mounted with ProLong Gold Antifade Mountant (Thermo Fisher Scientific).

**Cell staining.** The cells seeded on the microscope cover glass were fixed with 4% PFA for 20 min, then washed with PBS containing 0.1% Triton X-100 and blocked in 10% FBS for 1 h. The cells were incubated with the primary antibodies at 4 ℃ overnight and incubated with Alexa Fluor-conjugated secondary antibodies for 1 h at room temperature, then washed three times with PBS and finally mounted with ProLong Gold Antifade Mountant (Thermo Fisher Scientific). Images were acquired with a confocal microscope (Leica TCS SP8). Images were processed using ImageJ.

The following antibodies and dyes were used: mouse anti-c-Abl (Sigma; A5844), rabbit anti-αSMA (Abcam; ab184705), rabbit anti-Col1a1 (Cell Signaling Technology; #72,026), rabbit anti-PDGFRα (Cell Signaling Technology; #3174), and mouse Anti-RACK1 (BD Biosciences; 610,177), rabbit anti-Paxillin (Abways, P49023), rabbit anti-vinculin (Abways, CY5164), Stress fiber dyes (Beyotime Biotechnology, C2205S), α-Tubulin dyes (Beyotime Biotechnology, C1050). Donkey anti-Mouse IgG (H + L), Alexa Fluor™ Plus 594 (Thermo Fisher Scientific, A32744), Donkey anti-Rabbit IgG (H + L), Alexa Fluor™ Plus 488 (Thermo Fisher Scientific, A32790).

### Western blot analysis

Renal tissues or NRK-49F cells were homogenized and lysed in radioimmunoprecipitation buffer containing a Protease Inhibitor Cocktail (MedChemExpress; HY-K0010) and Phosphatase Inhibitor Cocktail II (MedChemExpress; HY-K0022). Protein concentration was determined by using a BCA Protein Assay Kit (Thermo Scientific™; 23,225). Equal amounts of protein were separated on SDS–polyacrylamide gels in a Tris/glycine buffer system, and transferred onto PVDF membranes, then blocked in QuickBlock™ buffer (Beyotime Biotechnology; P0220). The membranes were incubated with the primary antibodies overnight at 4 °C. Blots were developed by using HRP-conjugated anti-mouse or anti-rabbit IgG secondary antibodies (Proteintech; PR30011, PR30012) and Western Bright ECL HRP substrate (Advansta). Protein bands were imaged with a ChemiDoc MP system (Bio-Rad Laboratories). The intensity of the protein bands was quantified by applying ImageJ software.

The following antibodies were used: mouse anti-GAPDH (Proteintech; 60,004–1-Ig), mouse anti-c-Abl (Sigma; A5844), rabbit anti-α-SMA (Abcam; ab184705), rabbit anti-Col1a1 (Cell Signaling Technology; #72,026), rabbit anti-Vimentin (Abcam; ab92547), mouse anti-RACK1 (BD Biosciences; 610,177), mouse anti-Type I collagen (Proteintech, 66,761), rabbit anti-FAK (Abways, Q05397), rabbit anti-FAK (Abways, Q05397).

### Focal adhesion morphometrics and analysis

Adhesion analysis was carried out using ImageJ software. After the background is minimized, the local contrast of the image is enhanced by running CLAHE, after which the BRIGHTNESS and CONTRAST algorithms are automatically adjusted. The Execute ANALYZE PARTICLES command scans the thresholded image and finds the edges of objects or particles. size = 25-infinity and circularity = 0.00–0.99, and quantitative statistics were collected (e.g., size, area, number of focal adhesions, and % area occupied by focal adhesions).

### Gel contraction analysis

Fibroblast contractile activity was assessed by collagen contraction assays. Briefly, NRK-49F cells (5 × 10^4^ cells) were seeded into a 2 mg/ml gel matrix (Absin; abs47014921) containing high-concentration Corning collagen-I purified from the rat tail and cast into a 24-well plate. The gel contraction was monitored after 48 h by taking photographs of the gels. The percentage of gel contraction was quantified using the formula: percentage gel contraction = 100 × ((well area − gel area) ÷ well area)).

### Mass spectrometry

The total protein bound to c-Abl in the NRK-49F cells was extracted by coimmunoprecipitation. In brief, NRK-49F cells were treated with 10 ng/ml TGF-β1 (PeproTech, 100–21) for 24 h, with untreated cells serving as a control. Then, the cell lysate was incubated with mouse anti-c-Abl (Sigma; A5844) and beads. Protein digestion, labeling, and mass spectrometry analysis were completed in the instrumental analysis center of Shanghai Jiao Tong University. An LTQ-Orbitrap instrument (Thermo Fisher, USA) connected to a Nano ACQUITY UPLC system was used to analyze the labeled peptide samples as well as the acquired MS/MS spectra and parameters.

### Statistical analysis

All data are presented as the mean ± SEM, all experiments were replicated at least 3 times. GraphPad Prism in GraphPad Software (San Diego, CA) was used for all statistical analyses. Student’s t-test was conducted to determine the significance of differences between the two groups. *P* < 0.05 represented a statistically significant difference. The nonlinear least squares regression method is used for simple linear curve fitting. Other details, such as the number of replicates and level of significance, are mentioned in figure legends and supplementary tables. No samples or animals were excluded from the analysis. Sample size estimation was not performed, and sample size was determined by the number of animals in the colony of a determined age and gender.

## Results

### c-Abl is upregulated in mouse fibrotic kidneys and enriched in fibroblasts

To investigate the potential involvement of c-Abl in kidney fibrosis, we first assessed the expression level of c-Abl in fibrotic mouse kidney tissues induced by UUO. The expression of c-Abl was significantly upregulated in UUO kidneys but remained unchanged in contralateral (CL) kidneys (Figure S1A-C). Immunofluorescence (IF) staining further revealed that c-Abl was expressed at low levels in normal mouse renal interstitial cells but was highly colocalized with PDGFRα-positive fibroblasts in the fibrotic kidney tissues. PDGFRα-positive cells represent ECM-producing mesenchymal cells, including active fibroblasts and myofibroblasts, in the fibrotic kidneys of patients with CKD and UUO mice [19]. Additionally, c-Abl was coexpressed with αSMA^+^ and Col1a1^+^ myofibroblasts in UUO kidneys (Figure S1D). Overall, the increased expression of c-Abl in mouse fibrotic kidneys and its enrichment in ECM-producing PDGFRα^+^ mesenchymal cells and α-SMA^+^ myofibroblasts strongly suggest a potential role for c-Abl in the pathogenesis of renal fibrosis.

### c-Abl deletion in ECM-producing PDGFRα^+^ mesenchymal cells attenuate kidney fibrosis following UUO injury

**Fig. 1:**
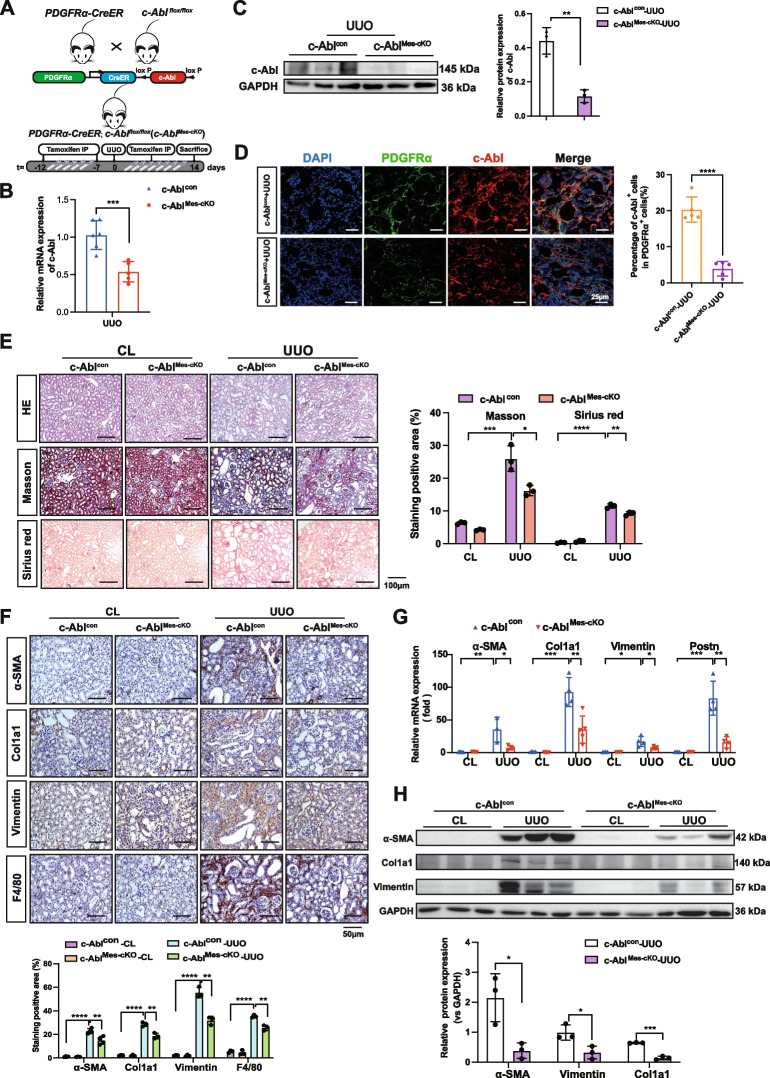
c-Abl deletion in ECM-producing PDGFRα^+^ mesenchymal cells attenuate kidney fibrosis following UUO injury. **A** Experimental design for inducing renal fibrosis in *c-Abl*^*Mes−cKO*^ and *c-Abl*^*con*^ mice. Kidneys were collected 14 days after UUO administration. **B** Quantitative RT‒PCR analysis of the *c-Abl* gene. **C** Western blot analysis and (**D**) IF staining of the c-Abl protein to detect its knockout efficiency in *c-Abl*^*Mes−cKO*^ UUO mice (*n* = 5) relative to that in *c-Abl*^*con*^ UUO mice (*n* = 6). Scale bar, 25 μm. GAPDH was used as a control. **E** H&E staining (top panel), Masson’s trichrome staining (middle panel), and Sirius red staining (bottom panel) of kidney sections from *c-Abl*^*Mes−cKO*^ and *c-Abl*^*con*^ mice 14 days after UUO administration. The results of quantitative analysis of Sirius red and Masson’s trichrome staining are shown (*n* ≥ 3). Scale bar, 100 μm. **F** Representative IHC images showing the downregulation of α-SMA, Col1a1, Vimentin, and F4/80 in *c-Abl*^*Mes−cKO*^ mice. The results of the quantitative analysis of the positive cells are shown. Scale bar, 50 μm. **G** Quantitative RT‒PCR analysis of *α-SMA*, *Col1a1*, *Vimentin,* and *Postn* and (**H**) Western blot analysis of α-SMA, Col1a1, and Vimentin were performed to detect extent of fibrosis in the kidneys of *c-Abl*^*Mes−cKO*^ UUO mice relative to those in the kidneys of *c-Abl*^*con*^ UUO mice (*n* ≥ 3). The data are presented as the mean ± SD. *P < 0.05, **P < 0.01, ***P < 0.001, ****P < 0.0001

**Fig. 2:**
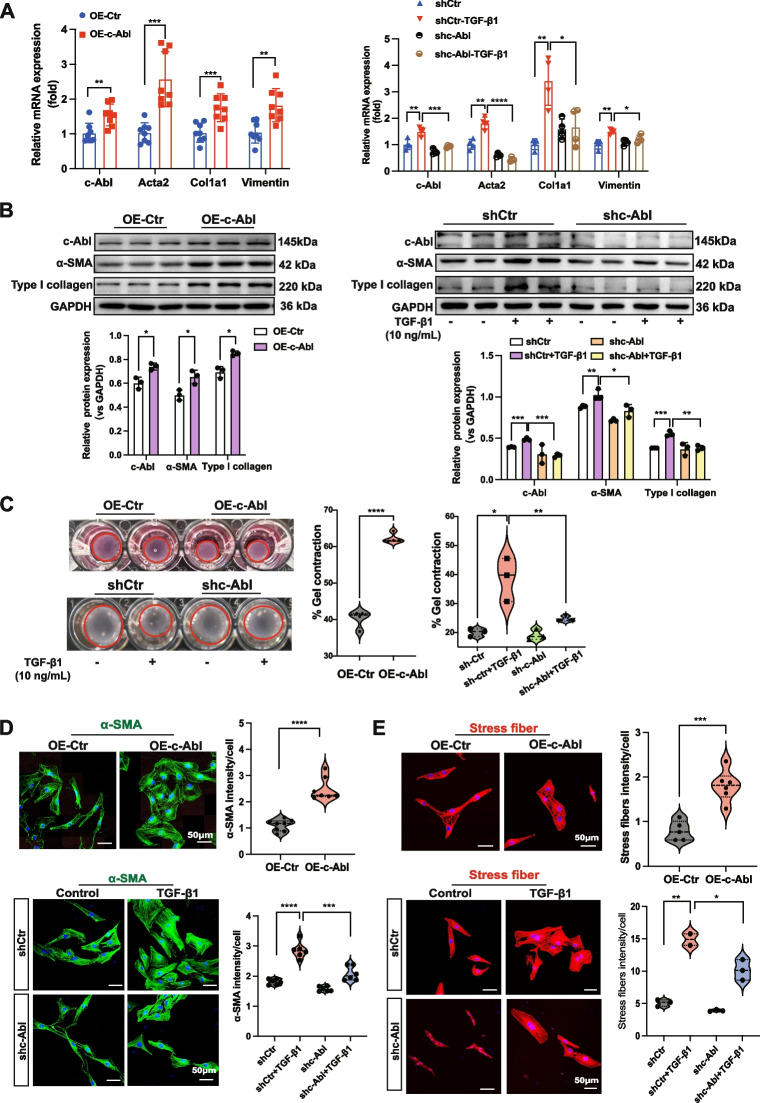
c-Abl is essential or inducing fibroblast activation, myofibroblast differentiation, and ECM production. **A** mRNA levels of the *c-Abl* gene and fibrotic genes (*Acta2, Col1a1,* and *vimentin*) and (**B**) protein levels (c-Abl, α-SMA, and type I collagen) in NRK-49F fibroblasts treated with TGF-β1 (10 ng/mL) for 24 h following transfection with the shc-Abl lentivirus or the c-Abl overexpression lentivirus for 48 h (*n* ≥ 3). **C** Representative images of Collagen contractility in NRK-49F fibroblasts transfected with the shc-Abl lentivirus or the c-Abl overexpression lentivirus for 48 h. The percentage of gel contraction was quantified using the formula: percentage gel contraction = 100 × ((well area − gel area) ÷ well area). An increase in the percentage of gel contraction indicates an increase in cell contraction. The data were obtained from three independent experiments. **D** Representative IF staining images of α-SMA and (**E**) stress fibers. The quantification of the average signal intensity for each cell (relative to the control) is included. The data were obtained from at least 3 independent biological samples per group in three independent experiments. Scale bars, 50 μm. The data are presented as the mean ± SD. *P < 0.05, **P < 0.01, ***P < 0.001, ****P < 0.0001

To gain insight into the role of c-Abl in regulating ECM-producing PDGFRα^+^ mesenchymal cells during renal fibrosis, an inducible knockout mouse model, *PDGFRα-CreER;c-Abl*^*flox/flox*^ (hereafter referred to as *c-Abl*^*Mes−cKO*^), was generated and subsequently administered UUO injury (Fig. 1A). *c-Abl*^*Mes−cKO*^ mice are viable, fertile, and visually indistinguishable from control mice (*c-Abl*^*flox/flox*^, referred to here as *c-Abl*^*con*^). The kidney morphology or histology of *c-Abl*^*Mes−cKO*^ did not differ from that of *c-Abl*^*con*^ mice. The efficiency of the c-Abl knockout was confirmed through mRNA and protein analyses (Figs. 1B–2D). Masson staining and Sirius red staining revealed a reduction in collagen deposition in the *c-Abl*^*Mes−cKO*^ mice (Fig. 1E). Notably, the expression of α-SMA, Col1a1, Vimentin, Postn and F4/80 were significantly lower in the kidneys of *c-Abl*^*Mes−cKO*^ mice than in those of *c-Abl*^*con*^ mice (Fig. 1F-H). Taken together, these findings suggest that the deletion of c-Abl in ECM-producing PDGFRα^+^ mesenchymal cells significantly attenuate the progression of kidney fibrosis.

### c-Abl is essential for inducing fibroblast activation, myofibroblast differentiation, and ECM production

PDGFRα^+^ mesenchymal cells in the fibrotic kidneys of mice consist of fibroblasts and myofibroblasts, and FMT is a crucial process in renal fibrosis. To investigate the role of c-Abl in FMT, we stimulated rat renal interstitial fibroblasts (NRK-49F) with TGF-β1 (10 ng/mL) and observed time-dependent upregulation of the c-Abl, α-SMA, and ECM proteins (Figure S2). Next, we infected NRK-49F fibroblasts with lentivirus carrying c-Abl and found that overexpression of c-Abl significantly increased the expression of α-SMA, type I collagen, and vimentin. Additionally, c-Abl overexpression promoted the contraction of collagen gel matrices, indicating enhanced fibroblast activation and differentiation (Fig. 2A-C). Notably, c-Abl overexpression also induced stress fiber formation, as evidenced by the increased density of α-SMA and phalloidin staining, which are commonly used markers of the myofibroblast phenotype (Fig. 2D and E). Conversely, knockdown of c-Abl attenuated FMT and the profibrotic effects induced by TGF-β1, accompanied by reduced levels of α-SMA, vimentin, and Col1a1 (Fig. 2A and B), as well as diminished contraction of collagen gel matrices, indicating a decrease in the acquisition of the myofibroblast phenotype (Fig. 2C). Additionally, c-Abl deficiency impaired stress fiber formation (Fig. 2D, E). Collectively, these findings demonstrate that c-Abl, which is downstream of TGF-β1, is both necessary and sufficient for promoting FMT and stress fiber formation.

### c-Abl is a positive regulator of focal adhesions and actin stress fibers

**Fig. 3:**
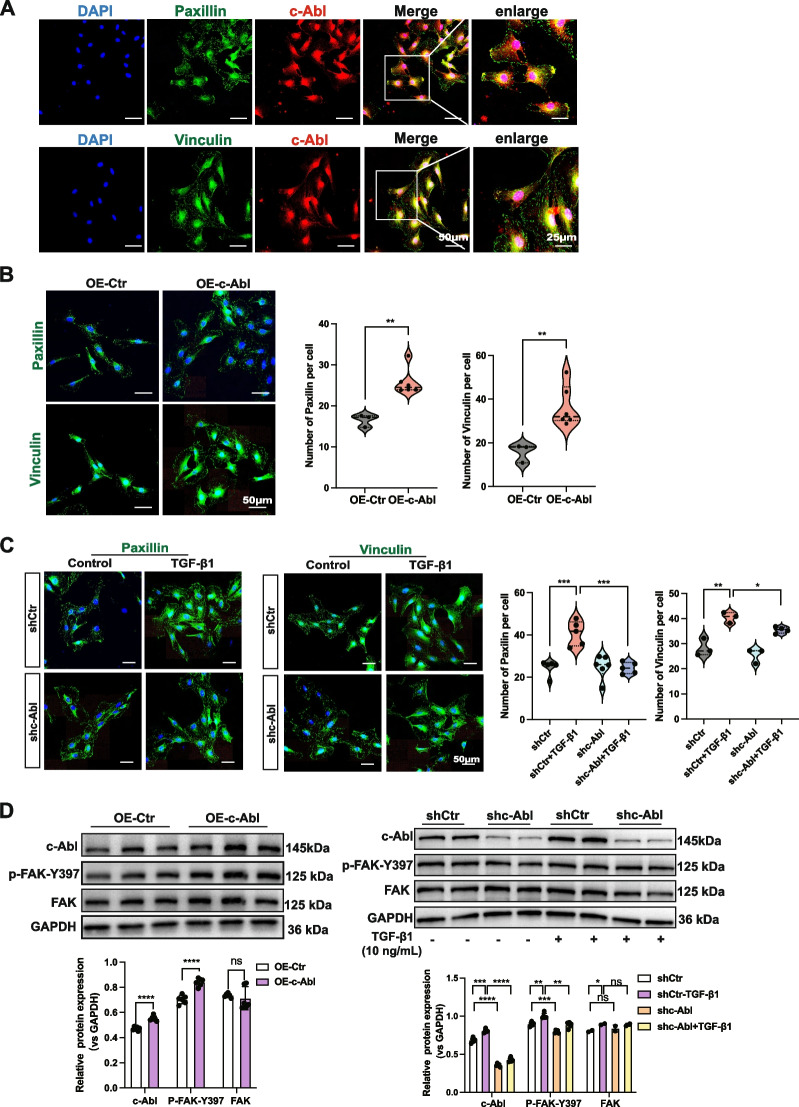
c-Abl is a positive regulator of focal adhesions and actin stress fibers. **A** Representative IF staining images showing the colocalization of c-Abl with paxillin or vinculin in NRK-49F fibroblasts treated with TGF-β1 (10 ng/mL) for 24 h. Scale bars, 50 μm. Enlarged images are shown on the left. Scale bars, 25 μm. **B** Representative IF images of paxillin and vinculin in NRK-49F fibroblasts transfected with the c-Abl overexpression lentivirus or empty lentivirus and (**C**) treated with TGF-β1 (10 ng/mL) for 24 h following transfection with the shc-Abl lentivirus or a control lentivirus for 48 h. Paxillin-positive or vinculin-positive focal adhesions were quantified as described in the Methods section (each data point represents one biological replicate). Scale bars, 50 μm. **D** FAK phosphorylation in c-Abl-overexpressing or (**E**) c-Abl-knockdown NRK-49F fibroblasts determined by Western blotting (*n* ≥ 3). GAPDH was used as a loading control. The data are presented as the mean ± SD. *P < 0.05, **P < 0.01, ***P < 0.001, ****P < 0.0001, ns = not significant

The assembly and formation of stress fibers are facilitated by focal adhesion maturation. As a result, we hypothesized that c-Abl could play a role in both focal adhesion maturation and stress fiber assembly. First, IF staining confirmed the colocalization of c-Abl with paxillin and vinculin, two protein markers of focal adhesions (Fig. 3A). Staining for paxillin and vinculin revealed an increased number of focal adhesions in the c-Abl-overexpressing cells and a decreased number in the c-Abl knockdown cells (Fig. 3B and C). Our subsequent examination of FAK phosphorylation at Y397, which regulates signal transmission from the ECM to the actin cytoskeleton [20], revealed that knocking down c-Abl in NRK-49F fibroblasts led to reduced FAK phosphorylation and c-Abl overexpression alone was sufficient to increase FAK phosphorylation (Fig. 3D). These findings suggest that c-Abl may be involved in focal adhesion maturation by regulating FAK activity.

### c-Abl maintains the stability of the RACK1 protein in fibroblasts

**Fig. 4:**
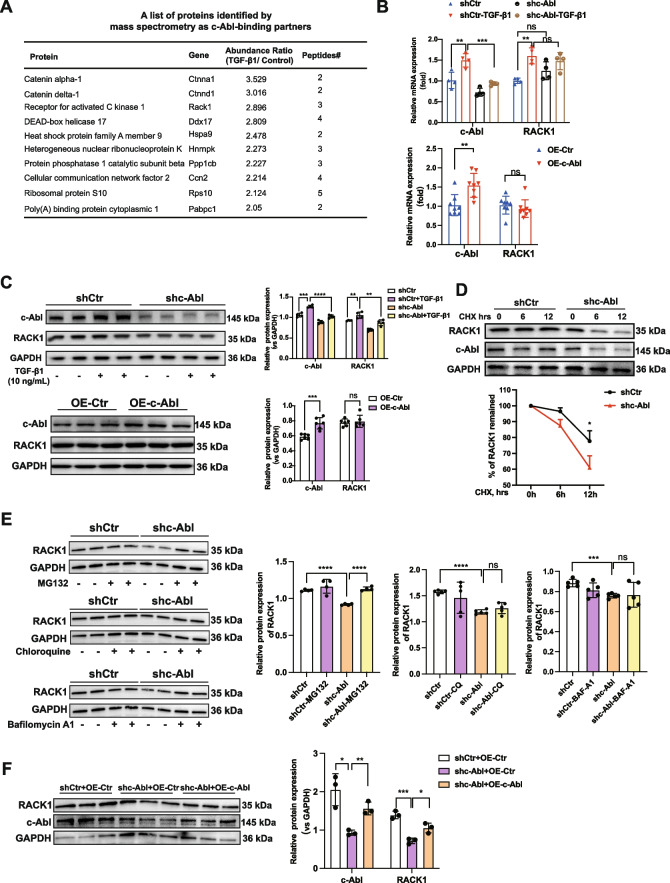
c-Abl maintains the stability of the RACK1 protein in renal fibroblasts. **A** Potential proteins identified as c-Abl-binding partners by mass spectrometry. **B** mRNA level of the *RACK1* gene and (**C**) protein level of RACK1 in NRK-49F fibroblasts treated with TGF-β1 (10 ng/mL) for 24 h following transfection with the shc-Abl lentivirus or a control lentivirus for 48 h or transfection with the c-Abl overexpression lentivirus or empty lentivirus for 48 h (*n* ≥ 3). GAPDH was used as a loading control. **D** Western blot analysis of RACK1 protein levels in c-Abl-knockdown NRK-49F fibroblasts treated with cycloheximide (CHX, 20 μg/ mL) for durations. **E** Detection of RACK1 protein stability in c-Abl-knockdown NRK-49F fibroblasts in the presence of the proteasome inhibitor MG132 (5 μM) or the lysosome inhibitors chloroquine (15 μM) and balfilomycin A1 (5 nM) for 10 h (*n* ≥ 3). GAPDH was used as a loading control. **F** The protein level of RACK1 in the c-Abl-knockdown NRK-49F fibroblasts followed by transfection with the c-Abl overexpression lentivirus or a control lentivirus. GAPDH was used as a loading control. The data are presented as the mean ± SD. *P < 0.05, **P < 0.01, ***P < 0.001, ****P < 0.0001, ns = not significant

To investigate the molecular mechanism underlying c-Abl-induced stress fiber formation and focal adhesion maturation, we employed liquid chromatography and tandem mass spectrometry (LC‒MS/MS) to screen for potential proteins that bind to c-Abl in NRK-49F cells treated with TGF-β1. Figure 4A shows that among the candidate partners, RACK1 was one of the most highly enriched proteins in the TGF-β1-treated group. Previous studies have highlighted the importance of RACK1 in FAK activity and focal adhesion maturation [21]. Overexpression of c-Abl in NRK-49F fibroblasts did not result in any changes in the mRNA or protein levels of RACK1. However, knocking down c-Abl significantly reduced the amount of RACK1 protein without affecting the mRNA levels (Fig. 4B and 4C). We speculated that c-Abl might contribute to the stability of RACK1 protein by preventing its degradation. We then performed a cycloheximide (CHX) assay and found that c-Abl deficiency remarkably accelerated RACK1 protein degradation in NRK-49F fibroblasts (Fig. 4D), indicating that c-Abl regulates RACK1 protein stability. Next, we found that MG132, a proteasome inhibitor but not a lysosome inhibitor such as chloroquine (CQ) or bafilomycin (BAF-A1), could notably reduce the degradation of the RACK1 protein in the absence of c-Abl, suggesting that RACK1 proteins undergo degradation through the proteasome pathway (Fig. 4E). Re-expression of c-Abl in c-Abl-knockdown NRK-49F fibroblasts effectively protected against the degradation of the RACK1 protein (Fig. 4F). These findings provide evidence that c-Abl plays a role in maintaining the stability of the RACK1 protein through inhibition of the proteasome system.

### RACK1 induces myofibroblast differentiation by increasing focal adhesions and actin stress fibers

Next, the expression of RACK1 was significantly increased in fibrotic kidneys compared to that in CL kidneys (Figure S3A-D) and was colocalized with PDGFRα^+^ mesenchymal cells and αSMA^+^ or Col1a1^+^ myofibroblasts in fibrotic kidneys (Figure S3D), implying that RACK1 may play a crucial role in FMT during renal fibrosis.

**Fig. 5:**
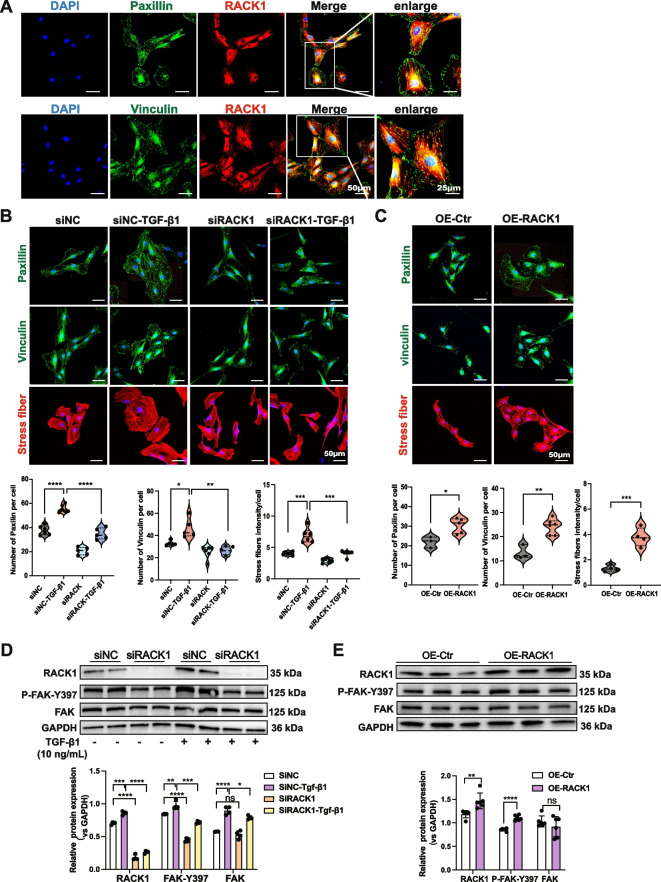
RACK1 increases the number of focal adhesions and actin stress fibers. **A** Representative IF staining images showing the colocalization of RACK1 with paxillin or vinculin in NRK-49F fibroblasts treated with TGF-β1 (10 ng/mL) for 24 h. Scale bars, 50 μm. The enlarged images represent the merged images shown on the left. Scale bars, 25 μm. **B** Representative IF staining images of paxillin, vinculin, and stress fibers in NRK-49F fibroblasts treated with TGF-β1 (10 ng/mL) for 24 h following transfection with siRACK1 or the siNC control for 24 h and (**C**) transfection with the RACK1 overexpression lentivirus or empty lentivirus for 48 h. Paxillin- or vinculin-containing focal adhesions were quantified (each data point represents one biological replicate). Scale bars, 50 μm. **D**, **E** The phosphorylation of FAK protein in NRK-49F fibroblasts treated with RACK1 overexpression or RACK1 silencing was determined by western blotting (*n* ≥ 3). GAPDH was used as a control. The data are presented as the mean ± SD. *P < 0.05, **P < 0.01, ***P < 0.001, ****P < 0.0001

In NRK-49F fibroblasts treated with TGF-β1, RACK1 colocalized with paxillin and vinculin in focal adhesions (Fig. 5A). Additionally, the number of focal adhesions and stress fibers was significantly decreased and increased in RACK1-knockdown and RACK1-overexpressing fibroblasts, respectively (Fig. 5B and C). As expected, phosphorylation of FAK at Y397 was reduced in RACK1-knockdown fibroblasts and increased in RACK1-overexpressing cells (Fig. 5D, E). These findings support the importance of RACK1 in focal adhesion formation in NRK-49F fibroblasts.

**Fig. 6:**
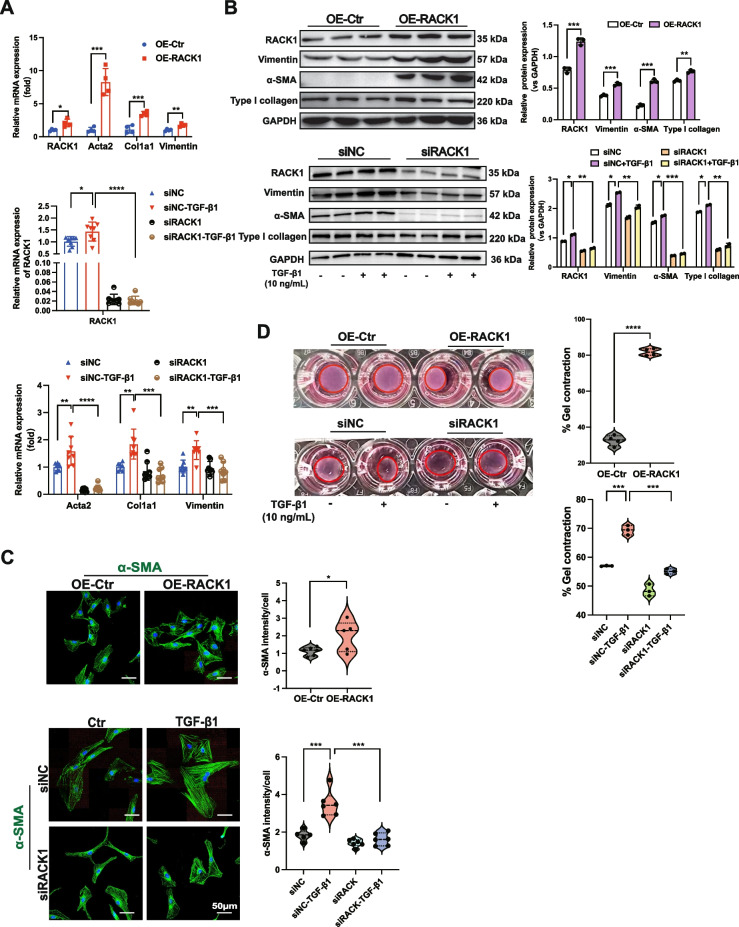
RACK1 induces fibroblast activation, myofibroblast differentiation, and ECM production. **A** mRNA levels of the *RACK1* gene and fibrotic genes (*Acta2, Col1a1,* and *vimentin*) and (**B**) protein levels (RACK1, α-SMA, vimentin, and type I collagen) in NRK-49F fibroblasts transfected with the RACK1 overexpression lentivirus for 48 h or treated with TGF-β1 (10 ng/mL) for 24 h following transfection with the siRACK1 or siNC control (*n* ≥ 3). GAPDH was used as a loading control. **C** Representative images of Collagen contractility in NRK-49F fibroblasts in the indicated groups. The data were obtained from three independent experiments. **D** Representative IF staining of α-SMA in the indicated groups (*n* ≥ 3). Scale bars, 50 μm. The data are presented as the mean ± SD. *P < 0.05, **P < 0.01, ***P < 0.001, ****P < 0.0001

Next, we investigated the function of RACK1 during FMT. Overexpression of RACK1 induced fibroblast activation and increased the expression of vimentin and collagen proteins, particularly α-SMA. Conversely, knocking down RACK1 reduced the expression of these genes (Fig. 6A - C). Furthermore, RACK1 overexpression promoted the contractility of collagen gel matrices, while inhibition of RACK1 expression reduced contractility (Fig. 6D). These results clearly demonstrated that RACK1 positively regulates FMT in renal fibroblasts, at least partially through increasing focal adhesions and actin stress fibers.

### c-Abl enhances myofibroblast differentiation and ECM deposition via the RACK1 protein

**Fig. 7:**
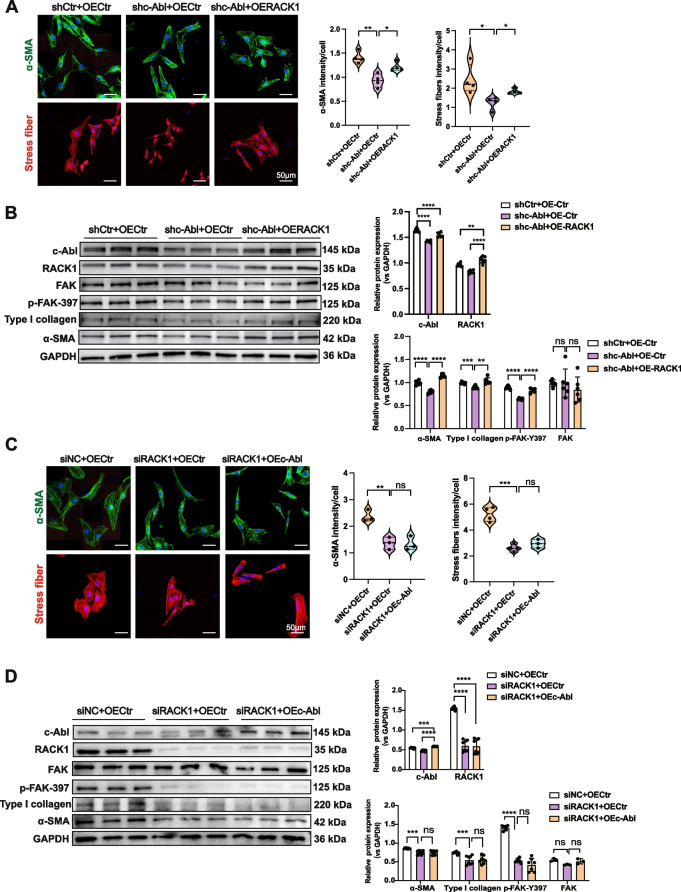
c-Abl enhances myofibroblast differentiation and ECM deposition via RACK1. **A** IF staining for α-SMA and stress fiber staining in NRK-49F fibroblasts transfected with the RACK1 overexpression lentivirus following treatment with the c-Abl siRNA. The quantification of the total signal intensity for each cell (relative to the control) is included. Scale bars, 50 μm. **B** The protein levels of c-Abl, RACK1, FAK, and renal fibrosis-related proteins (α-SMA and type I collagen) were detected in the indicated groups (*n* ≥ 3). GAPDH was used as a loading control. **C** IF staining for α-SMA and stress fiber staining of NRK-49F fibroblasts transfected with the c-Abl overexpression lentivirus following treatment with RACK1 siRNA. The quantification of the total signal intensity for each cell (relative to the control) is included. Scale bars, 50 μm. **D** The protein levels of c-Abl, RACK1, FAK, and fibrosis-related proteins (α-SMA and type I collagen) were detected in the indicated groups (*n* ≥ 3). GAPDH was used as a loading control. The data are presented as the mean ± SD. *P < 0.05, **P < 0.01, ***P < 0.001, ****P < 0.0001, ns = not significant

To further understand the regulatory effect of c-Abl on FMT mediated by RACK1, RACK1 was overexpressed in c-Abl-silenced NRK-49F fibroblasts. The results showed that RACK1 overexpression partially restored impaired stress fiber formation, as quantified by α-SMA and phalloidin staining (Fig. 7A). This increased stress fiber formation subsequently promoted the phosphorylation of downstream FAK and the expression of type I collagen and α-SMA (Fig. 7B). Importantly, overexpression of c-Abl in the absence of RACK1 did not increase the expression of fibrosis-related markers such as α-SMA or type I collagen, nor did it promote the phosphorylation of FAK or the assembly of stress fibers (Fig. 7C and D). Unexpectedly, in c-Abl knockdown NRK-49F fibroblasts, overexpression of RACK1 was accompanied by a significant up-regulation of c-Abl at both transcriptional and protein levels (Figure S4). We speculated that RACK1 may promote FMT, thereby exacerbating the renal fibrotic process. This process indirectly upregulates c-Abl expression, creating a positive c-Abl-RACK1 feedback loop in the FMT process. Overall, these results demonstrated that RACK1 is indispensable for FMT induced by c-Abl.

### c-Abl induces myofibroblast differentiation by promoting microtubule depolymerization

In addition to focal adhesions, the depolymerization of microtubules can also induce stress fiber assembly and promote cell contraction [22]. The overexpression of c-Abl led to the reduction of tubulin staining and the depolymerization of the microtubular network, which was prevented by paclitaxel, a microtubule stabilizer (Figure S5A). Conversely, the knockdown of c-Abl enhanced the formation of the microtubular network, which was attenuated by vinblastine, an inhibitor of microtubule polymerization (Figure S5A).

Next, we investigated whether c-Abl might regulate stress fiber formation and FMT via modulation of the microtubular network. Indeed, the stabilization of microtubules by paclitaxel reduced the FMT induced by c-Abl overexpression, as evidenced by decreased collagen production and α-SMA expression (Figure S5B and C). Notably, stress fiber formation was significantly reduced, indicating a decreased connection between the ECM and the actin cytoskeleton (Figure S5D). However, the depolymerization of microtubules by vinblastine abolished the inhibitory effects of c-Abl knockdown on TGFβ-induced FMT (Figure S5B, C and E). Thus, c-Abl induces stress fiber assembly, FMT, and collagen deposition not only by promoting focal adhesion maturation but also by promoting microtubule depolymerization.

### c-Abl ablation in renal myofibroblasts decreased the progression of established kidney fibrosis

**Fig. 8:**
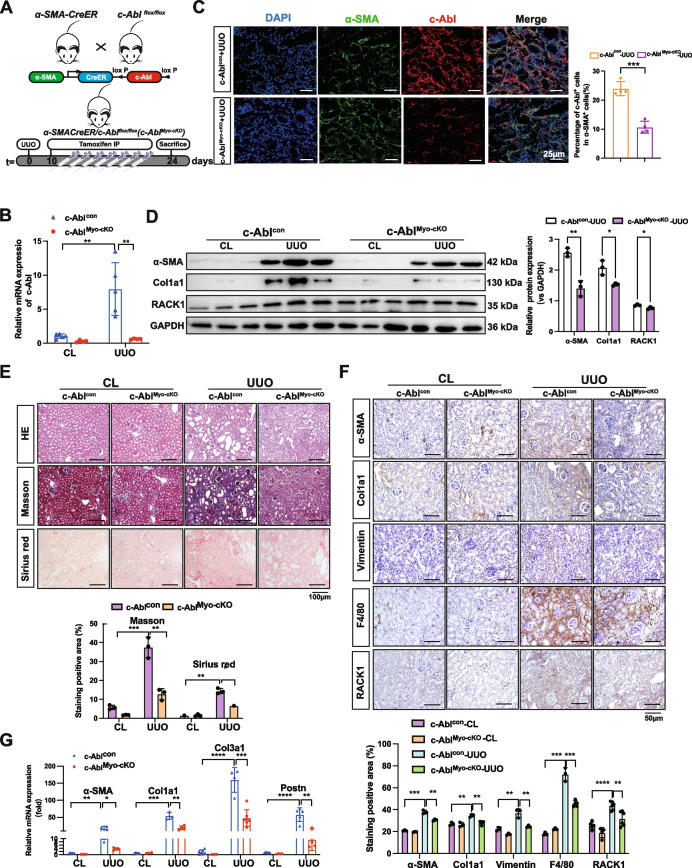
c-Abl ablation in renal myofibroblasts decreased the progression of established kidney fibrosis. **A** Experimental design for inducing renal fibrosis in *c-Abl*^*Myo−cKO*^ and *c-Abl*^*con*^ mice. **B** Quantitative RT‒PCR analysis of the *c-Abl* gene and **C** IF staining of c-Abl to detect the knockout efficiency in *c-Abl*^*Myo−cKO*^ UUO mice (*n* ≥ 3). Scale bar, 25 μm. **D** Western blot analysis of RACK1, α-SMA, and Col1a1 was performed to detect the extent of fibrosis in the kidneys of *c-Abl*^*Myo−cKO*^ UUO mice relative to those in the kidneys of *c-Abl*^*con*^ UUO mice (*n* ≥ 3). GAPDH was used as a loading control. **E** H&E staining (top panel), Masson’s trichrome staining (middle panel), and Sirius red staining (bottom panel) of kidney sections from *c-Abl*^*Myo−cKO*^ and *c-Abl*^*con*^ mice after UUO administration. The results of quantitative analysis of Sirius red and Masson’s trichrome staining are shown (*n* ≥ 3). Scale bar, 100 μm. **F** Representative IHC images showing the downregulation of α-SMA, Col1a1, Vimentin, and F4/80 in *c-Abl*^*Myo−cKO*^ mice. The results of the quantitative analysis of the positive cells are shown. Scale bar, 50 μm. **G** Quantitative RT‒PCR analysis of α-SMA, Col1a1, Col3a1, and Postn was performed to detect the extent of fibrosis in the kidneys of *c-Abl*.^*Myo−cKO*^ UUO mice (*n* ≥ 3). The data are presented as the mean ± SD. *P < 0.05, **P < 0.01, ***P < 0.001, ****P < 0.0001

To assess the potential benefits of specifically targeting c-Abl in renal myofibroblasts in established renal fibrosis, we generated an inducible, myofibroblast-specific knockout strain (*α-SMA-CreER;c-Abl*^*flox/flox*^*, c-Abl*^*Myo−cKO*^) and administered tamoxifen 10 days after UUO. At this time point, extensive renal fibrosis was observed (Fig. 8A). *c-Abl*^*Myo−cKO*^ mice are viable, fertile, and visually indistinguishable from *c-Abl*^*con*^ mice. The kidney morphology or histology of *c-Abl*^*Myo−cKO*^ did not differ from that of *c-Abl*^*con*^ mice. The expression levels of c-Abl were effectively reduced in *c-Abl*^*Myo−cKO*^ mice (Fig. 8B, C). As expected, myofibroblast-specific c-Abl deficiency resulted in the downregulation of RACK1, α-SMA, and Col1a1 protein expression in UUO-treated *c-Abl*^*Myo−cKO*^ mouse kidneys (Fig. 8D). The accumulation and deposition of total collagen were significantly attenuated in the injured kidneys of the *c-Abl*^*Myo−cKO*^ mice compared to those of the *c-Abl*^*con*^ mice (Fig. 8E). Additionally, IHC staining of fibrotic markers revealed decreases in the kidneys of the *c-Abl*^*Myo−cKO*^ mice following UUO treatment (Fig. 8F). The mRNA levels of α-SMA, Col1a1, Col3a1, and Postn were also reduced in the *c-Abl*^*Myo−cKO*^ mice, demonstrating that specific knockout of c-Abl in myofibroblasts effectively alleviated established renal fibrosis (Fig. 8G). These findings suggest that in vivo targeting of c-Abl may be a novel and potent therapeutic approach for ameliorating kidney fibrosis and slowing the progression of CKD.

### High expression of c-Abl in patients with CKD is positively correlated with RACK1 and fibrotic risk factors

**Fig. 9:**
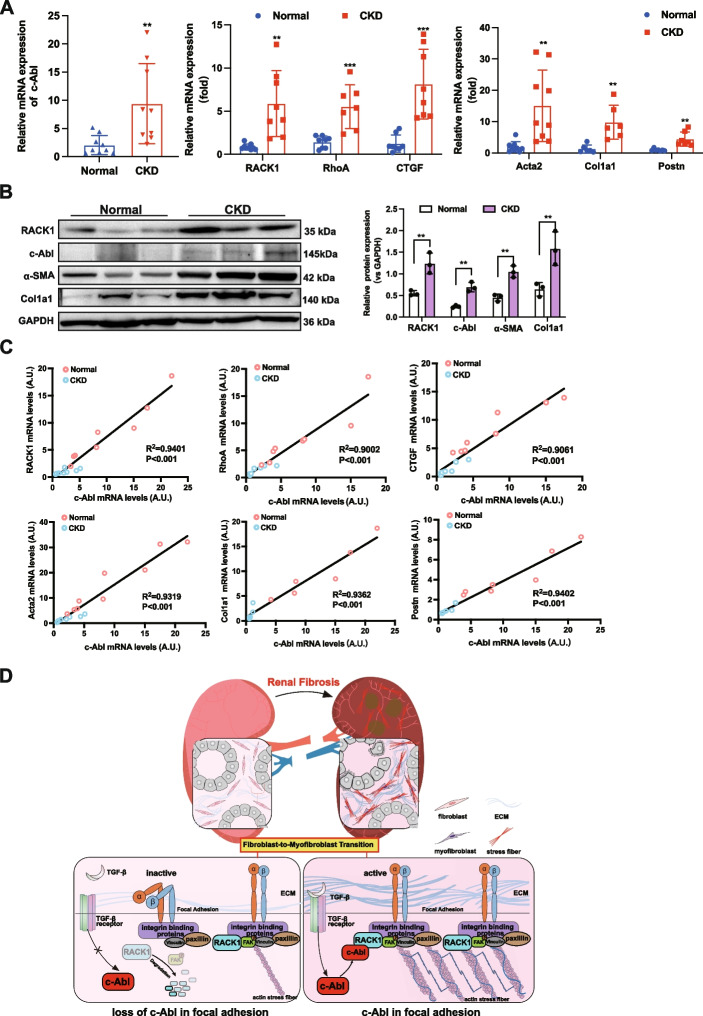
High expression of c-Abl in patients with CKD is positively correlated with RACK1 and other fibrotic risk factors. **A** mRNA levels of c-Abl and RACK1; the cytoskeletal remodeling-related genes RhoA and CTGF; and the ECM-related genes Acta2, Col1a1, and Postn determined by quantitative RT‒PCR in kidney tissues from healthy donors (*n* = 9) and patients with CKD (*n* = 9). **B** Western blot analysis of c-Abl, RACK1, and fibrosis-related proteins (Col1a1 and α-SMA) in healthy donors and patients with CKD (*n* ≥ 3). GAPDH was used as a loading control. **C** Linear regression revealing a positive correlation between c-Abl expression and the expression of RACK1 or characteristic cytoskeletal remodeling-related genes (CTGF and RhoA) and ECM-related genes (Acta2, Col1a1, and Postn). **D** Schematic image of the role of c-Abl in the FMT process during renal fibrosis. Briefly, in the injured renal mesenchyme, activation of the noncanonical TGF-β signaling pathway results in upregulation of the downstream tyrosine kinase c-Abl, which subsequently binds to the RACK1 protein and maintains its stability. RACK1, as a scaffold protein, recruits the c-Abl and integrin binding-related proteins FAK, paxillin, and vinculin to focal adhesions to induce the assembly of stress fibers, cell contraction, and fibroblast activation, which increases ECM production. Combined with TGF-β1 and the ability of focal adhesions to sense ECM mechanical force, c-Abl further promoted the differentiation of myofibroblasts during FMT. Thus, the c-Abl-RACK1-FAK signaling axis exacerbates fibroblast activation, myofibroblast differentiation, and ECM deposition, ultimately resulting in renal fibrosis

We next examined the expression of c-Abl in kidney samples from 9 patients with uremia and CKD who underwent nephrectomy and in healthy kidneys from 9 donors without CKD. The mRNA levels of *c-Abl* and *RACK1* were significantly greater in the kidneys of patients with CKD than in those of healthy subjects (Fig. 9A). Additionally, the expression of markers of cytoskeleton-associated genes (*RhoA* and *CTGF*) and ECM-related genes (*Acta2, Col1a1, and Postn*) was significantly upregulated in CKD kidneys (Fig. 9A). The protein levels of c-Abl, RACK1, and ECM marker genes were markedly elevated in patients with CKD but not in healthy individuals (Fig. 9B). The expression of c-Abl showed a positive linear correlation with RACK1, RhoA, CTGF, α-SMA, Col1a1, and Postn levels in CKD kidneys (Fig. 9C). In conclusion, the upregulation of c-Abl in human fibrotic kidneys and its positive correlation with RACK1 and focal adhesion-associated genes support the use of c-Abl as a potential therapeutic target in kidney fibrosis.

## Discussion

c-Abl has been documented to play a role in multiorgan fibrosis, specifically renal fibrosis [23, 24]. Among the key cell types involved in renal fibrosis are myofibroblasts, which serve as the primary source of ECM. Fibroblasts undergo activation and differentiation into myofibroblasts through FMT. Nonetheless, the involvement of c-Abl in FMT and the underlying molecular mechanism by which c-Abl regulates this process have not been fully elucidated. In our investigation, we observed heightened expression of c-Abl in renal interstitial cells, such as fibroblasts and myofibroblasts, in injured kidneys. Furthermore, specific deletion of c-Abl in ECM-producing PDGFRα^+^ mesenchymal cells significantly mitigated renal fibrosis. Mechanistically, c-Abl triggered the formation of stress fibers and cell contraction, thereby promoting the FMT process by maintaining the stability of the RACK1 protein, which scaffolds c-Abl and FKA at focal adhesions (Fig. 9D). Ultimately, the removal of c-Abl from myofibroblasts was found to be beneficial in established kidney fibrosis, suggesting that targeting c-Abl could be a potent therapeutic strategy for mitigating kidney fibrosis and decelerating the progression of CKD.

Fibroblasts can undergo FMT upon injury, during which they express a substantial amount of ECM proteins. Dysregulation and excessive production of ECM proteins are known to cause fibrosis, such as renal fibrosis, in damaged tissues [25]. Abl kinases are widely recognized for their evolutionarily conserved and well-characterized role in regulating cytoskeletal dynamics, which leads to cellular shape changes and migration. These kinases also play crucial roles in cellular stress responses and morphogenesis, particularly in epithelial-mesenchymal transition [26, 27]. However, the specific mechanism by which c-Abl regulates FMT remains largely unclear. To address this knowledge gap, we conducted a mass spectrometry investigation to identify potential downstream targets of c-Abl during FMT. Notably, our analysis revealed that the RACK1 protein is among the top-ranked targets and is highly likely to be phosphorylated by c-Abl. RACK1, a multifunctional scaffolding protein, is highly conserved and expressed in various cellular compartments, orchestrating a range of fundamental cellular activities, such as proliferation, differentiation, and protein synthesis [28]. Acting as a signaling hub, RACK1 recruits active enzymes and partner proteins through its seven WD repeats, which adopt a propeller structure [29] with multiple protein-binding sites facilitating interactions with partner proteins harboring docking modules such as C2 domains (PKCs) and SH2 domains (Src and Fyn) [16, 21]. Our findings suggest that RACK1 may bind to c-Abl, which possesses an SH2 domain preceding its tyrosine kinase domain [30]. Unfortunately, our attempts to detect the binding of these proteins through conventional Co-IP experiments were unsuccessful. We hypothesize that the weak binding force between c-Abl and RACK1 may account for this difficulty, a supposition supported by another study demonstrating that the interaction between c-Abl and RACK1 becomes more evident in the presence of insulin-like growth factor[31]. Curiously, we observed that c-Abl is necessary for maintaining RACK1 protein stability but not for maintaining RACK1 mRNA transcription. In the absence of c-Abl, the RACK1 protein undergoes degradation via the proteasome pathway. The ability of RACK1 to coordinate signaling events relies heavily on the stoichiometric ratio between scaffold proteins and their interacting partners, implying that the stability or expression level of RACK1 significantly impacts numerous cellular processes. Indeed, aberrant RACK1 expression has been implicated in disorders such as Alzheimer's disease [32] and cancer [33]. On the basis of our findings, we propose that c-Abl promotes renal fibrosis through the maintenance of RACK1 protein stability. Interestingly, we observed that the expression of c-Abl were regulated by RACK1. As a scaffold protein, RACK1 is unlikely to directly regulate the transcription of the c-Abl gene and is more likely to indirectly regulate its expression through other ways. We speculated that RACK1 may promote FMT, thereby exacerbating the renal fibrotic process. The fibrotic microenvironment indirectly upregulates c-Abl expression. Conversely, increased c-Abl expression will enhance the stability of RACK1 protein, thereby creating a positive feedback loop in the FMT process.

Subsequently, we demonstrated that RACK1 is indispensable for c-Abl-induced renal fibrosis by promoting FAK activity and FMT progression. Our data and prior studies have established that RACK1 enhances focal adhesion maturation and stress fiber assembly [34–36]. Furthermore, the phosphorylation of RACK1 at tyrosine 52 by c-Abl facilitates its interaction with FAK [37], which is crucial for cell adhesion and migration. Collectively, our findings suggest that the c-Abl-RACK1-FAK signaling axis promotes FMT by inducing focal adhesion maturation and stress fiber assembly, representing a novel molecular mechanism underlying the pathogenesis of renal fibrosis.

The assembly of stress fibers and myofibroblast differentiation are associated not only with focal adhesions but also with the state of microtubules. Generally, disruption of the microtubule network promotes the formation and maturation of focal adhesions, subsequently stimulating the assembly of actin stress fibers and facilitating fibroblast-to-myofibroblast transition [22, 38–41]. It is currently unknown whether c-Abl contains microtubule-binding domains [30], but our results revealed that c-Abl can induce microtubule depolymerization in renal fibroblasts and subsequently promote FMT. Therefore, c-Abl may regulate this process in various ways, including by regulating focal adhesion formation and stress fiber assembly through the c-Abl-RACK1-FAK signaling axis or by inducing stress fiber assembly through direct promotion of microtubule depolymerization. Both mechanisms ultimately contribute to fibroblast activation, myofibroblast differentiation, and renal fibrosis. Recently, researchers have focused on the common links between the c-Abl-mediated regulation of structural changes involving the actin cytoskeleton and organelle dynamics and the transcriptional program activated during synaptic plasticity, with the aim of finding potentially effective treatments to improve degenerative results and delay memory loss in neurodegenerative diseases [42–44]. This further supports the importance of c-Abl in cytoskeletal remodeling and its therapeutic implications for disease.

Our findings demonstrated that knocking out c-Abl not only in PDGFRα-positive ECM-producing cells (*c-Abl*^*Mes−cKO*^ mice) but also specifically in α-SMA-positive myofibroblasts (*c-Abl*^*Myo−cKO*^ mice), effectively alleviated renal fibrosis, which indicated that on the one hand, α-SMA positive myofibroblasts are the main cell type responsible for renal fibrosis. On the other hand, even if renal fibrosis progresses to the mature stage, knocking out c-Abl still can effectively alleviate fibrosis, indicating its potential as a therapeutic target. Additionally, we observed high expression levels of c-Abl in the kidneys of CKD patients, which showed a positive linear correlation with the expression of cytoskeletal remodeling-related genes (RACK1, RhoA, and CTGF). These findings suggest that targeting c-Abl could be a novel and powerful therapeutic approach for ameliorating kidney fibrosis and slowing the progression of CKD.

In conclusion, our study revealed a significant and previously unrecognized novel link between the nonreceptor tyrosine kinase c-Abl and the pathogenesis of renal fibrosis, particularly during the FMT process. By elucidating the mechanisms through which the c-Abl/RACK1/FAK pathway regulates the FMT process and ECM deposition, our study has provided further evidence that c-Abl is a potential therapeutic target for treating CDK and renal fibrosis, particularly in patients with c-Abl overexpression and/or activation.

### Supplementary Information

**Supplementary Material 1.**

## Data Availability

No datasets were generated or analysed during the current study.

## Abbreviations

CKD: Chronic kidney disease
FMT: Fibroblast-myofibroblast transition
ECM: Extracellular matrix
FAK: Focal adhesion kinase
TGF-β1: Transforming growth factor-beta 1
UUO: Unilateral ureteral obstruction
RACK1: Receptor for activated C-kinase 1
CL: Contralateral

## References

[1] Humphreys BD (2018). Mechanisms of Renal Fibrosis. Annu Rev Physiol.

[2] Mills KT, Xu Y, Zhang W, Bundy JD, Chen CS, Kelly TN, Chen J, He J (2015). A systematic analysis of worldwide population-based data on the global burden of chronic kidney disease in 2010. Kidney Int.

[3] Breyer MD, Susztak K (2016). The next generation of therapeutics for chronic kidney disease. Nat Rev Drug Discov.

[4] Tojkander S, Gateva G, Lappalainen P (2012). Actin stress fibers–assembly, dynamics and biological roles. J Cell Sci.

[5] Johnson CP, Tang HY, Carag C, Speicher DW, Discher DE (2007). Forced unfolding of proteins within cells. Science.

[6] Mimura Y, Ihn H, Jinnin M, Asano Y, Yamane K, Tamaki K (2005). Constitutive phosphorylation of focal adhesion kinase is involved in the myofibroblast differentiation of scleroderma fibroblasts. J Invest Dermatol.

[7] Meng XM, Nikolic-Paterson DJ, Lan HY (2016). TGF-β: the master regulator of fibrosis. Nat Rev Nephrol.

[8] Tang PM, Zhang YY, Mak TS, Tang PC, Huang XR, Lan HY (2018). Transforming growth factor-β signalling in renal fibrosis: from Smads to non-coding RNAs. J Physiol.

[9] Santibañez JF, Quintanilla M, Bernabeu C (2011). TGF-β/TGF-β receptor system and its role in physiological and pathological conditions. Clin Sci (Lond).

[10] Mora AL, Rojas M, Pardo A, Selman M (2017). Emerging therapies for idiopathic pulmonary fibrosis, a progressive age-related disease. Nat Rev Drug Discov.

[11] Herbertz S, Sawyer JS, Stauber AJ, Gueorguieva I, Driscoll KE, Estrem ST, Cleverly AL, Desaiah D, Guba SC, Benhadji KA (2015). Clinical development of galunisertib (LY2157299 monohydrate), a small molecule inhibitor of transforming growth factor-beta signaling pathway. Drug Des Devel Ther.

[12] Anderton MJ, Mellor HR, Bell A, Sadler C, Pass M, Powell S, Steele SJ, Roberts RR, Heier A (2011). Induction of heart valve lesions by small-molecule ALK5 inhibitors. Toxicol Pathol.

[13] Karimizadeh E, Gharibdoost F, Motamed N, Jafarinejad-Farsangi S, Jamshidi A, Mahmoudi M (2015). c-Abl silencing reduced the inhibitory effects of TGF-β1 on apoptosis in systemic sclerosis dermal fibroblasts. Mol Cell Biochem.

[14] Lu Y, Lv F, Kong M, Chen X, Duan Y, Chen X, Sun D, Fang M, Xu Y (2019). A cAbl-MRTF-A Feedback Loop Contributes to Hepatic Stellate Cell Activation. Front Cell Dev Biol.

[15] Bibi Y, Gottlieb AB (2008). A potential role for imatinib and other small molecule tyrosine kinase inhibitors in the treatment of systemic and localized sclerosis. J Am Acad Dermatol.

[16] Adams DR, Ron D, Kiely PA (2011). RACK1, A multifaceted scaffolding protein: Structure and function. Cell Commun Signal.

[17] Wendling O, Bornert JM, Chambon P, Metzger D (2009). Efficient temporally-controlled targeted mutagenesis in smooth muscle cells of the adult mouse. Genesis.

[18] Kua HY, Liu H, Leong WF, Li L, Jia D, Ma G, Hu Y, Wang X, Chau JF, Chen YG (2012). c-Abl promotes osteoblast expansion by differentially regulating canonical and non-canonical BMP pathways and p16INK4a expression. Nat Cell Biol.

[19] Kuppe C, Ibrahim MM, Kranz J, Zhang X, Ziegler S, Perales-Patón J, Jansen J, Reimer KC, Smith JR, Dobie R (2021). Decoding myofibroblast origins in human kidney fibrosis. Nature.

[20] Parsons JT, Martin KH, Slack JK, Taylor JM, Weed SA (2000). Focal adhesion kinase: a regulator of focal adhesion dynamics and cell movement. Oncogene.

[21] Duff D, Long A (2017). Roles for RACK1 in cancer cell migration and invasion. Cell Signal.

[22] Ng DH, Humphries JD, Byron A, Millon-Frémillon A, Humphries MJ (2014). Microtubule-dependent modulation of adhesion complex composition. PLoS ONE.

[23] Rosenbloom J, Castro SV, Jimenez SA (2010). Narrative review: fibrotic diseases: cellular and molecular mechanisms and novel therapies. Ann Intern Med.

[24] Wang S, Wilkes MC, Leof EB, Hirschberg R (2010). Noncanonical TGF-beta pathways, mTORC1 and Abl, in renal interstitial fibrogenesis. Am J Physiol Renal Physiol.

[25] Sun C, Tian X, Jia Y, Yang M, Li Y, Fernig DG (2022). Functions of exogenous FGF signals in regulation of fibroblast to myofibroblast differentiation and extracellular matrix protein expression. Open Biol.

[26] Greuber EK, Smith-Pearson P, Wang J, Pendergast AM (2013). Role of ABL family kinases in cancer: from leukaemia to solid tumours. Nat Rev Cancer.

[27] Bradley WD, Koleske AJ (2009). Regulation of cell migration and morphogenesis by Abl-family kinases: emerging mechanisms and physiological contexts. J Cell Sci.

[28] Gandin V, Senft D, Topisirovic I, Ronai ZA (2013). RACK1 Function in Cell Motility and Protein Synthesis. Genes Cancer.

[29] McCahill A, Warwicker J, Bolger GB, Houslay MD, Yarwood SJ (2002). The RACK1 scaffold protein: a dynamic cog in cell response mechanisms. Mol Pharmacol.

[30] Hernández SE, Krishnaswami M, Miller AL, Koleske AJ (2004). How do Abl family kinases regulate cell shape and movement?. Trends Cell Biol.

[31] Khanna RS, Le HT, Wang J, Fung TC, Pallen CJ (2015). The interaction of protein-tyrosine phosphatase α (PTPα) and RACK1 protein enables insulin-like growth factor 1 (IGF-1)-stimulated Abl-dependent and -independent tyrosine phosphorylation of PTPα. J Biol Chem.

[32] Battaini F, Pascale A (2005). Protein kinase C signal transduction regulation in physiological and pathological aging. Ann N Y Acad Sci.

[33] Al-Reefy S, Osman H, Jiang W, Mokbel K (2010). Evidence for a pro-apoptotic function of RACK1 in human breast cancer. Oncogene.

[34] Hermanto U, Zong CS, Li W, Wang LH (2002). RACK1, an insulin-like growth factor I (IGF-I) receptor-interacting protein, modulates IGF-I-dependent integrin signaling and promotes cell spreading and contact with extracellular matrix. Mol Cell Biol.

[35] Zhang G, Gao Z, Guo X, Ma R, Wang X, Zhou P, et al. CAP2 promotes gastric cancer metastasis by mediating the interaction between tumor cells and tumor-associated macrophages. J Clin Invest. 2023;133(21):e166224. 10.1172/JCI166224 PMC10617780 37707957

[36] Vomastek T, Iwanicki MP, Schaeffer HJ, Tarcsafalvi A, Parsons JT, Weber MJ (2007). RACK1 targets the extracellular signal-regulated kinase/mitogen-activated protein kinase pathway to link integrin engagement with focal adhesion disassembly and cell motility. Mol Cell Biol.

[37] Kiely PA, Baillie GS, Barrett R, Buckley DA, Adams DR, Houslay MD, O'Connor R (2009). Phosphorylation of RACK1 on tyrosine 52 by c-Abl is required for insulin-like growth factor I-mediated regulation of focal adhesion kinase. J Biol Chem.

[38] Bershadsky A, Chausovsky A, Becker E, Lyubimova A, Geiger B (1996). Involvement of microtubules in the control of adhesion-dependent signal transduction. Curr Biol.

[39] Liu BP, Chrzanowska-Wodnicka M, Burridge K (1998). Microtubule depolymerization induces stress fibers, focal adhesions, and DNA synthesis via the GTP-binding protein Rho. Cell Adhes Commun.

[40] Hinz B, Dugina V, Ballestrem C, Wehrle-Haller B, Chaponnier C (2003). Alpha-smooth muscle actin is crucial for focal adhesion maturation in myofibroblasts. Mol Biol Cell.

[41] Hinz B, Phan SH, Thannickal VJ, Prunotto M, Desmoulière A, Varga J, De Wever O, Mareel M, Gabbiani G (2012). Recent developments in myofibroblast biology: paradigms for connective tissue remodeling. Am J Pathol.

[42] Gutiérrez DA, Chandía-Cristi A, Yáñez MJ, Zanlungo S, Álvarez AR (2023). c-Abl kinase at the crossroads of healthy synaptic remodeling and synaptic dysfunction in neurodegenerative diseases. Neural Regen Res.

[43] Motaln H, Rogelj B (2023). The role of c-Abl tyrosine Kinase in brain and its pathologies. Cells.

[44] Lindholm D, Pham DD, Cascone A, Eriksson O, Wennerberg K, Saarma M (2016). c-Abl Inhibitors Enable Insights into the Pathophysiology and Neuroprotection in Parkinson's Disease. Front Aging Neurosci.

