# Supplementary material for: The c-Abl-RACK1-FAK signaling axis promotes renal fibrosis in mice through regulating fibroblast-myofibroblast transition
Source: Cell Commun Signal. 2024 Apr 30;22:247. doi: 10.1186/s12964-024-01603-z (PMC11059681; doi:10.1186/s12964-024-01603-z)
Supplement: Supplementary file 1 — Supplementary Material 1. [file 12964_2024_1603_MOESM1_ESM.pdf]

SUPPLEMENTARY MATERIALS

Supplementary Methods

Animals

c-Abl$^{loxP/loxP}$ (abbreviated as *c-Abl*^{con}) mice were crossed with *PDGFRα-CreER* mice or with *α-SMA-CreER* mice to generate tamoxifen-inducible, mesenchymal-specific and myofibroblast-specific knockout strain (abbreviated as *c-Abl*$^{Mes\text{-}cKO}$ and *c-Abl*$^{Myo\text{-}cKO}$), respectively. Offspring were bred to homozygosity to be used for subsequent studies. Only male animals were used. Genotyping PCR primers are provided in **supplementary table S1**.

We initially targeted 8-10 animals per group per time point for post-UUO analyses. The actual number of animals used for each experiment varied with breeding productivity at the time of surgery. Cage locations were randomly assigned in the housing facility. Animals were randomly ordered for surgery or euthanasia, but operators were aware of the set of animal genotypes included each day. Genotypes were unblinded for subsequent analyses after euthanasia.

Animals were used at 8 weeks of age. For temporal deletion of c-Abl, *c-Abl*$^{Mes\text{-}cKO}$ adult male mice were injected intraperitoneally with 20mg/ml tamoxifen 150ul (Sigma-Aldrich, T5648) for 5 consecutive days one week before UUO surgery, then tamoxifen was consecutively administered for 7 consecutive days after UUO to ensure the deletion of c-Abl in newly generated fibroblasts. *c-Abl*$^{Myo\text{-}cKO}$ adult male mice were administered tamoxifen 150ul every two days for 14 days after 10 days with UUO surgery, a time

point when extensive renal fibrosis happened.

UUO. Male mice (*c-Abl*^{con}, *c-Abl*$^{Mes\text{-}cKO}$, and *c-Abl*$^{Myo\text{-}cKO}$ mice) were anesthetized with an intraperitoneal injection of tribromoethanol. The abdomen of each mouse was opened, and the left ureter was ligated with 5-0 silk. The abdomen was then closed with running sutures, and the skin was closed with interrupted sutures. Body temperature was maintained at 38 °C throughout the procedure using an infrared homeothermic control system. The contralateral kidney was not manipulated.

After surgery, intraperitoneal (IP) warm sterile saline was provided to compensate for fluid loss during surgery. Analgesia with carprofen (5 μg/g IP) was provided for 48 h postoperatively (exactly 2 doses, immediately and 24 h after induction). After surgery, the mice were maintained in a temperature-controlled room under a 12 h light/dark cycle and reared on standard chow and water ad libitum. The surgery was maintained for 14 days. Animals with surgical complications were excluded from the analysis. Animals were euthanized using carbon dioxide at times indicated.

For 14d (without contralateral nephrectomy) UUO or uIRI surgery, there was no unexplained mortality owing to the short duration of follow up and preserved renal function provided by the intact contralateral kidney. All mice were randomly divided into different groups as indicated. All of the animal samples were analyzed in a blinded fashion.

Supplementary Figures

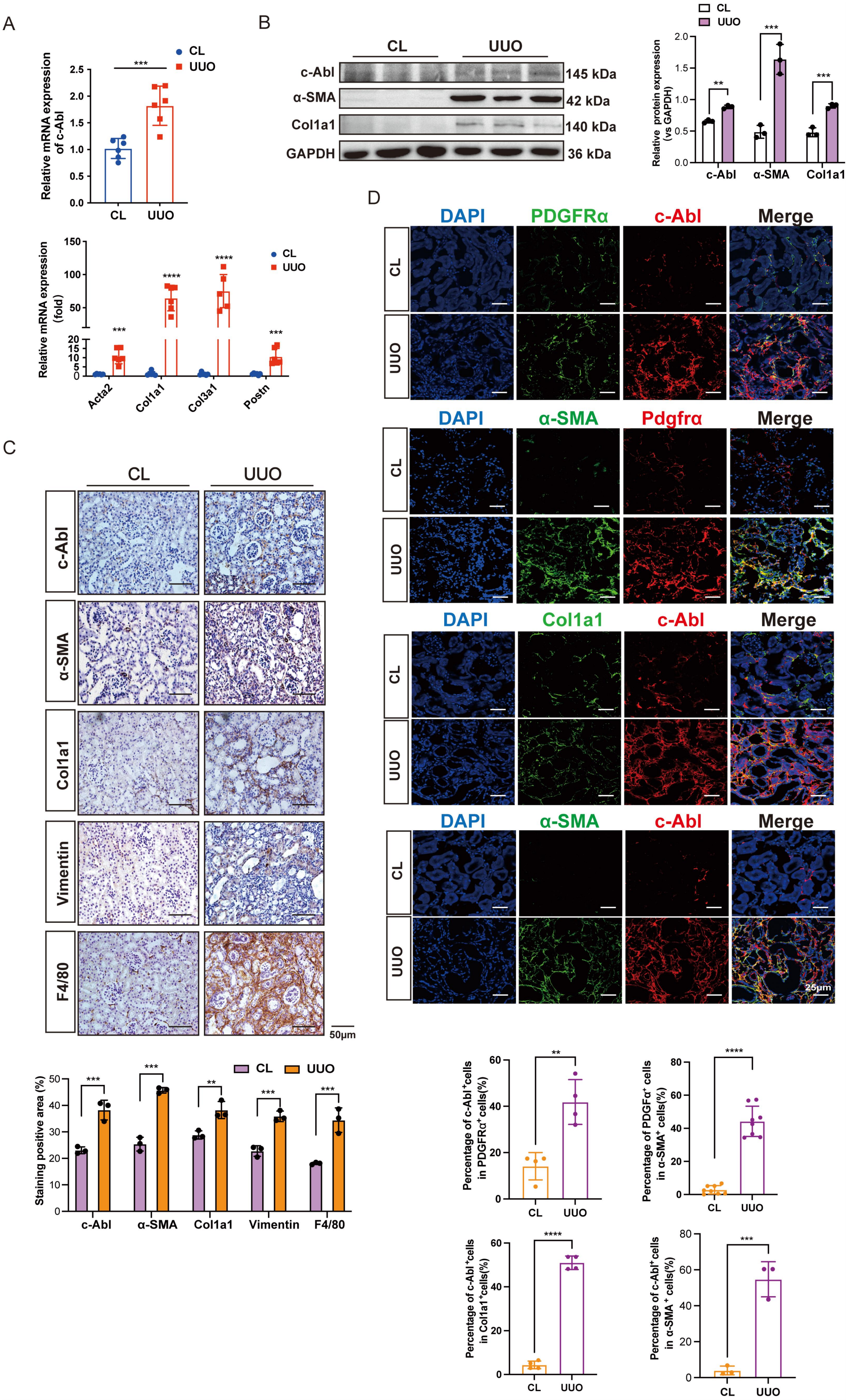

Supplementary Figure S1. c-Abl is upregulated in mouse fibrotic kidneys and enriched in fibroblasts. (A) Quantitative RT–PCR analysis of the *c-Abl, Acta2, Col1a1, Col3a1,* and *Postn* in mouse fibrotic kidneys induced by UUO kidneys compared with that in contralateral (CL) kidneys ($n \geq 5$). (B) Western blot analysis of c-Abl and renal fibrosis-related proteins (α-SMA and Col1a1) in whole-kidney lysates from WT mice 14 days after UUO ($n = 3$). GAPDH was used as a loading control. (C) Representative IHC images showing the upregulation of c-Abl, α-SMA, Col1a1, Vimentin, and F4/80 in UUO kidneys. The results of the quantitative analysis of the positive cells are shown. Scale bar, 50 μm. (D) Representative IF images showing the colocalization of PDGFRα with c-Abl, PDGFRα with α-SMA, and Col1a1 or α-SMA with c-Abl in the UUO or CL kidneys. The results of the quantitative analysis of the positive cells are shown. Cell nuclei were stained with DAPI. Scale bar, 25 μm. The data are presented as the mean ± SD. $^{**}P < 0.01$, $^{***}P < 0.001$, $^{****}P < 0.0001$.

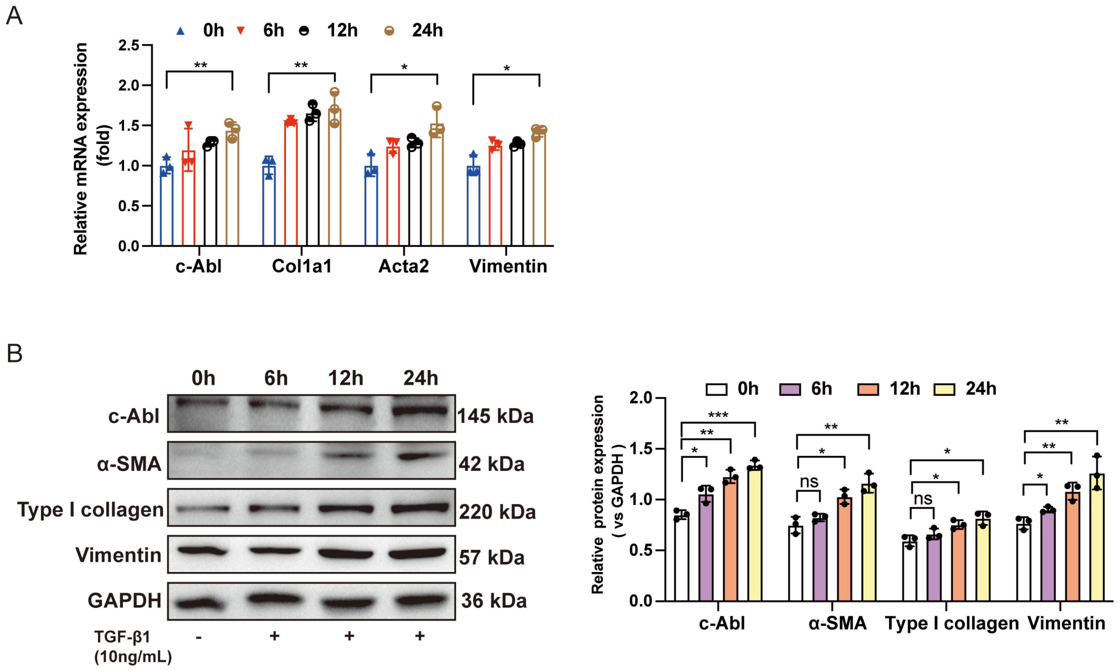

Supplementary Figure S2. TGF-β1 induces c-Abl expression in NRK-49F fibroblasts. (A) mRNA levels of the *c-Abl* gene and the fibrotic genes (*Col1a1, Acta2,* and *Vimentin*) in NRK-49F fibroblasts treated with TGF-β1 (10 ng/mL) for the indicated duration ($n \geq 3$). (B) Protein levels of c-Abl and the fibrotic proteins (α-SMA, Type I collagen, and Vimentin) in NRK-49F fibroblasts treated with TGF-β1 (10 ng/mL) for the indicated duration ($n \geq 3$). GAPDH was used as a loading control. The data are presented as the mean ± SD. $^{*}P < 0.05$, $^{**}P < 0.01$, ns = not significant.

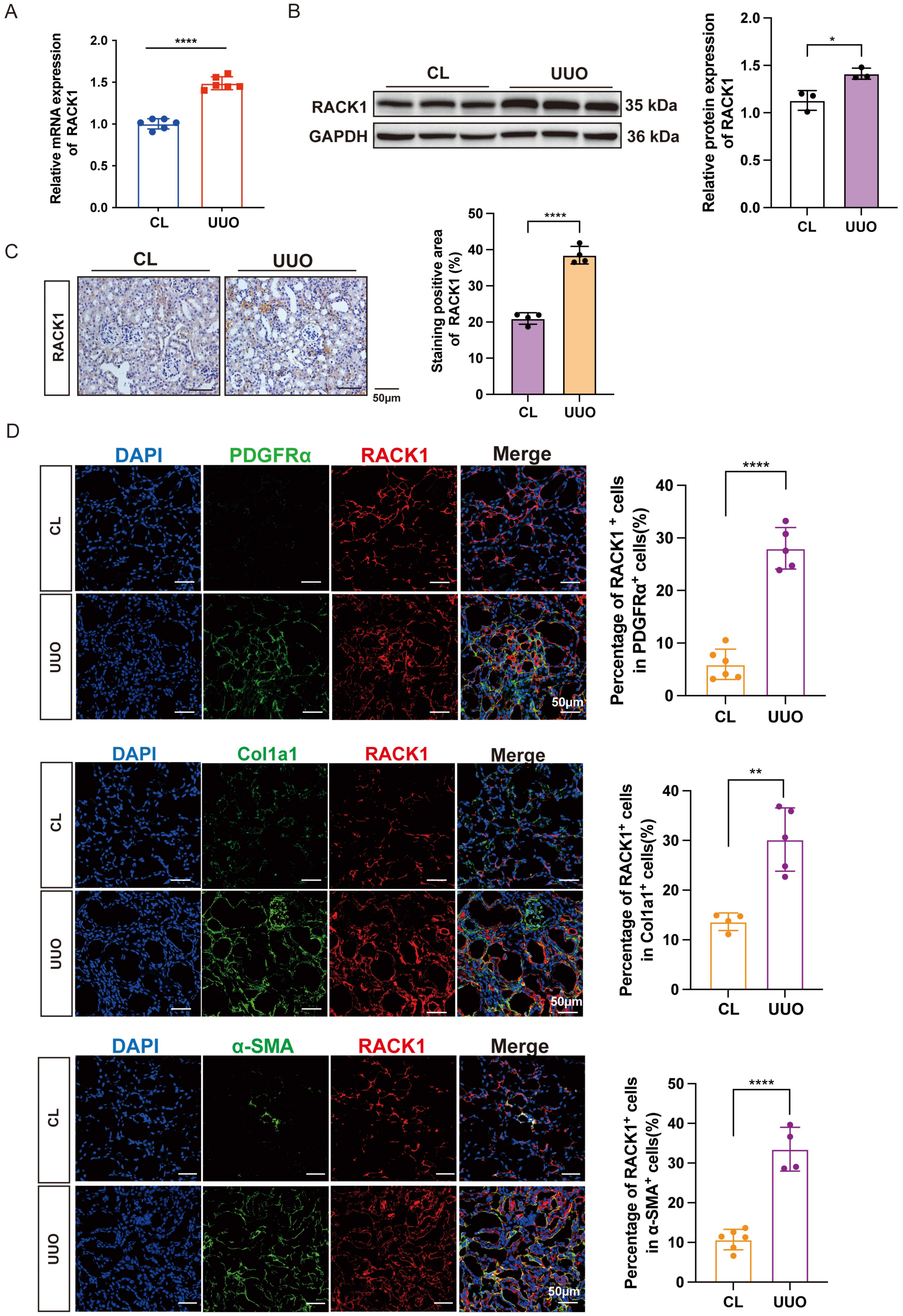

A
Relative mRNA expression of RACK1
****
CL
UUO
B
CL
UUO
RACK1
GAPDH
35 kDa
36 kDa
Relative protein expression of RACK1
*
C
RACK1
50μm
Staining positive area of RACK1 (%)
D
DAPI
PDGFRα
RACK1
Merge
Percentage of RACK1 + cells in PDGFRα+ cells(%)
Col1a1
Percentage of RACK1+ cells in Col1a1+ cells(%)
**
α-SMA
Percentage of RACK1+ cells in α-SMA+ cells(%)

Supplementary Figure S3. RACK1 is upregulated in mouse fibrotic kidneys and enriched in fibroblasts. (A) mRNA level, (B) protein level and (C) representative IHC images of the RACK1 in UUO kidneys compared with that in CL kidneys ($n \geq 3$). GAPDH was used as a loading control. Scale bar, 50 μm. (D) Representative IF images showing the colocalization of RACK1 with PDGFRα, Col1a1, and α-SMA in UUO or CL kidneys. The results of the quantitative analysis of the positive cells are shown. Cell nuclei were stained with DAPI. Scale bar, 50 μm. The data are presented as the mean ± SD. *$P < 0.05$, **$P < 0.01$, ****$P < 0.0001$.

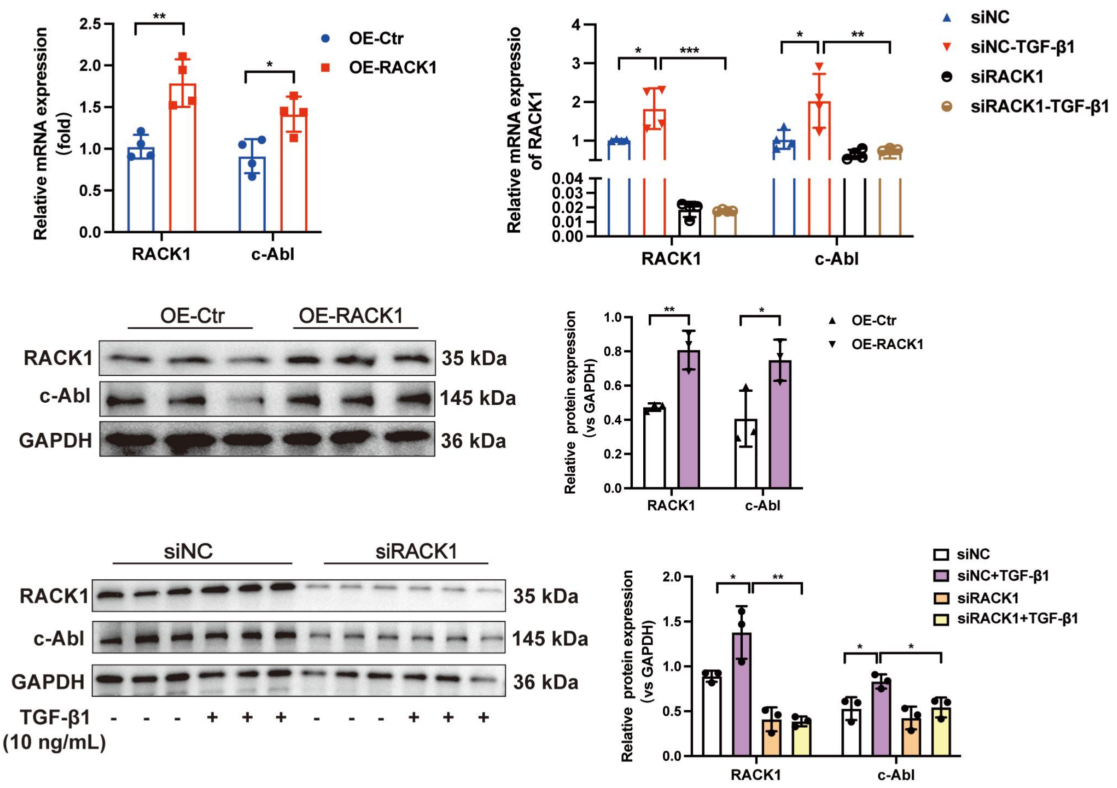

Supplementary Figure S4. RACK1 promoted the expression of c-Abl in both transcription and protein levels in NRK-49F fibroblasts treated with TGF-β1 following transfection with the siRACK1 or the RACK1 overexpression lentivirus (n ≥ 3).

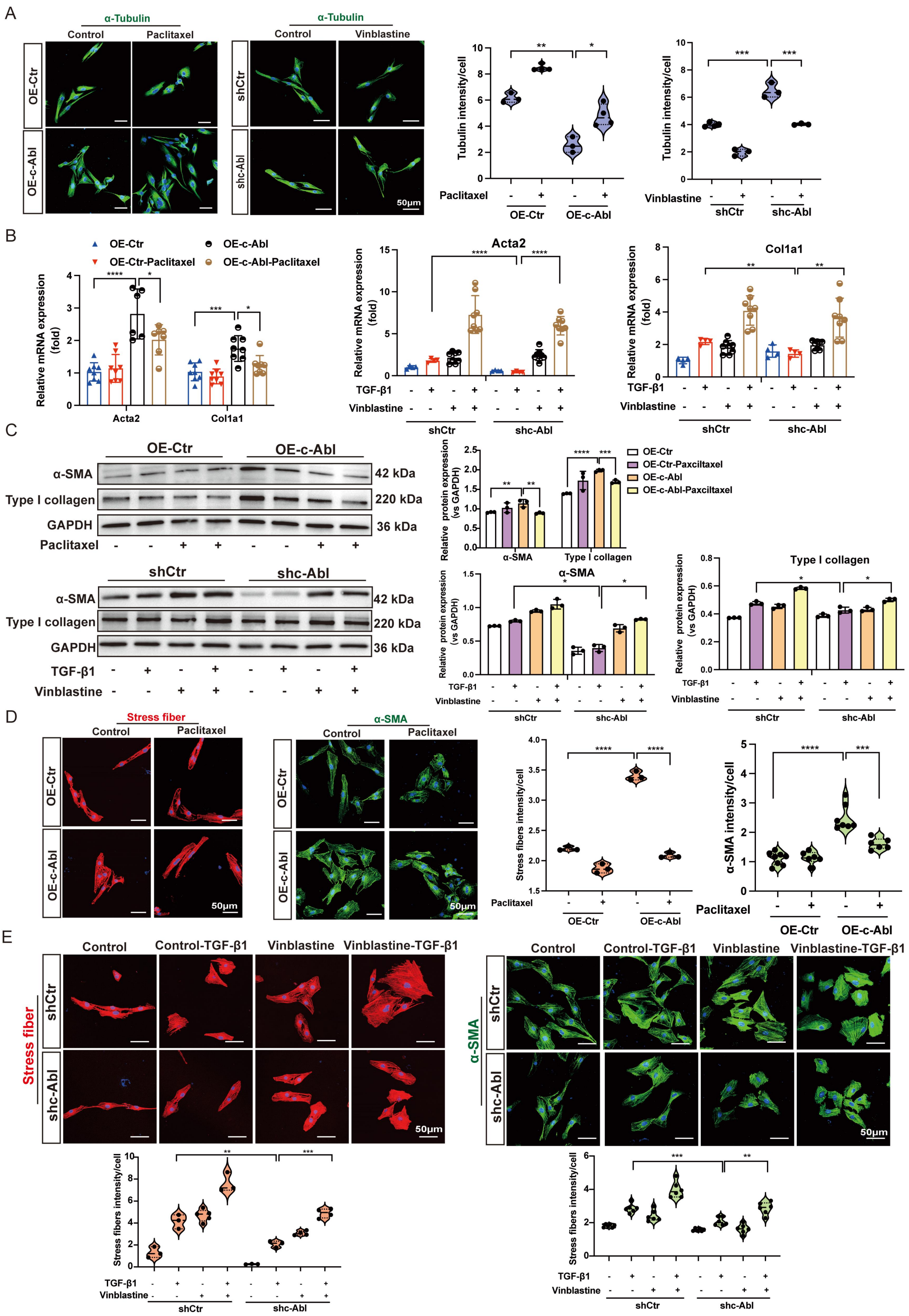

A
α-Tubulin
Control
Paclitaxel
Vinblastine
OE-Ctr
OE-c-Abl
shCtr
shc-Abl
50μm
Tubulin intensity/cell
B
OE-Ctr
OE-Ctr-Paclitaxel
OE-c-Abl
OE-c-Abl-Paclitaxel
Relative mRNA expression (fold)
Acta2
Col1a1
TGF-β1
Vinblastine
C
α-SMA
Type I collagen
GAPDH
42 kDa
220 kDa
36 kDa
Relative protein expression (vs GAPDH)
OE-Ctr-Paxciltaxel
OE-c-Abl-Paxciltaxel
D
Stress fiber
Stress fibers intensity/cell
α-SMA intensity/cell
E
Control-TGF-β1
Vinblastine-TGF-β1

Supplementary Figure S5. c-Abl induces myofibroblast differentiation by promoting microtubule depolymerization. (A) Representative IF staining images of α-Tubulin in NRK-49F fibroblasts transfected with the shc-Abl lentivirus or the c-Abl overexpression lentivirus for 36 h, then with paclitaxel (0.5 μM) or vinblastine (0.1μM) treatment for 12 h. The quantification of the total signal intensity for each cell (relative to the control) is included. Scale bars, 50 μm. (B) mRNA levels of fibrotic genes (*Acta2 and Col1a1*) and (C) protein levels of α-SMA and type I collagen in NRK-49F fibroblasts treated with TGF-β1 (10 ng/mL) following transfection with the shc-Abl lentivirus or the c-Abl overexpression lentivirus, then treated with paclitaxel or vinblastine for 12 hours. GAPDH was used as a loading control. (D, E) Representative IF staining images of α-SMA and stress fibers in the indicated groups. Quantification of the total signal intensity for each cell (relative to control) is included. Scale bars, 50 μm. The data are presented as the mean ± SD. $*P < 0.05$, $**P < 0.01$, $***P < 0.001$, $****P < 0.0001$.

Supplementary Table 1. Genotyping PCR Primers

	Primer Sequence (5' to 3')	
Target	Forward Primer	Reverse Primer
**tdTomato-WT**	AAGGGAGCTGCAGTGGAGTA	CCGAAAATCTGTGGGAAGTC
**tdTomato-Mut**	GGCATTAAAGCAGCGTATCC	CTGTTCCTGTACGGCATGG
**α-SMA-CreER-WT**	TTACGTCCATCGTGGACAGC	TGGGCTGGGTGTTAGCCTTA
**α-SMA-CreER-Mut**	ATTTGCCTGCATTACCGGTC	ATCAACGTTTTCTTTTCGGA
**PDGFRα-CreER-WT**	CAAATGTTGCTTGTCTGGTG	GTCAGTCGAGTGCACAGTTT
**PDGFRα-CreER-Mut**	TCAGCCTTAAGCTGGGACAT	ATGTTTAGCTGGCCCAAATG
**c-Abl flox**	AGGCCTTCTTCCTGATAGTC	CAGCAACCGGCTTGCATG

Supplementary Table 2. Nucleotide sequence of the primers used for qRT-PCR

Nucleotide sequence of the human primer used for qRT-PCR

	Primer Sequence (5' to 3')	
Gene	Forward Primer	Reverse Primer
**c-Abl**	5'-AAGCCGCTCGTTGGAACTC-3'	5'-AGACCCGGAGCTTTTCACCT-3'
**ROCK**	5'-AACATGCTGCTGGATAAATCTGG-3'	5'-TGTATCACATCGTACCATGCCT-3'
**RhoA**	5'-GGAAAGCAGGTAGAGTTGGCT-3'	5'-GGCTGTCGATGGAAAAACACA-3'
**CTGF**	5'- CAGCATGGACGTTCGTCTG-3'	5'-AACCACGGTTTGGTCCTTGG-3'
**RACK1**	5'-AGCAGCAACCCTATCATCGTC-3'	5'-TGAGATCCCATAACATGGCCT-3'
**Acta2**	5'-GTGTTGCCCCTGAAGAGCAT-3'	5'-GCTGGGACATTGAAAGTCTCA-3'
**Col1a1**	5'- GAGGGCCAAGACGAAGACATC-3'	5'-CAGATCACGTCATCGCACAAC-3'
**Postn**	5'-CTCATAGTCGTATCAGGGGTCG-3'	5'-ACACAGTCGTTTTCTGTCCAC-3'
**18S**	5'-CATTCGAACGTCTGCCCTATC-3'	5'-CCTGCTGCCTTCCTTGGA-3'

Nucleotide sequence of the mouse primer used for qRT-PCR

	Primer Sequence (5' to 3')	
Gene	Forward Primer	Reverse Primer
c-Abl	5'-AACACCCTAACCTGGTGCAG-3'	5'-GTGTGGCCATGTAGAGCAGT-3'
Col1a1	5'-GCTCCTCTTAGGGGCCACT-3'	5'-CCACGTCTCACCATTGGGG-3'
Acta2	5'-AAGAGGAAGACAGCACAGCC-3'	5'-AGCGTCAGGATCCCTCTCTT-3'
Vimentin	5'-TGGCACGTCTTGACCTTGAA-3'	5'-AGGTCAGGCTTGGAAACGTC-3'
Col3a1	5'-CTGTAACATGGAAACTGGGGAAA-3'	5'-CCATAGCTGAACTGAAAACCACC-3'
Postn	5'-CCTGCCCTTATATGCTCTGCT-3'	5'-AAACATGGTCAATAGGCATCACT-3'
RACK1	5'-AGGGCCACAATGGATGGGTA-3'	5'-CTGGTCAGCTTCCACATGATG-3'

Nucleotide sequence of the rat primer used for qRT-PCR

	Primer Sequence (5' to 3')	
Gene	Forward Primer	Reverse Primer
**c-Abl**	5'-AAGGAAGCCGCAGTGATGAA-3'	5'-GCAGTGGTGATACAGCAGGT-3'
**Col1a1**	5'-CAGATTGAGAACATCCGCAGC-3'	5'-CGGAACCTTCGCTTCCATACTC-3'
**Acta2**	5'-CATCCGACCTTGCTAACGGA-3'	5'-AGTCCAGAGCGACATAGCAC-3'
**Vimentin**	5'-CGCCACCTTCGTGAATACC-3'	5'-ACCGTCTTAATCAGGAGTGTTCT-3'
**α-SMA**	5'-CATCCGACCTTGCTAACGGA-3'	5'-GTCCAGAGCGACATAGCACA-3'
**RACK1**	5'-AGCAAGAAGTTATCAGCACCAG-3'	5'-CCAATAGTCACCTGCCATACA-3'
**GAPDH**	5'-GGAGTCTACTGGCGTCTTCAC-3'	5'-ATGAGCCCTTCCACGATGC-3'
